# The patterned assembly and stepwise Vps4-mediated disassembly of composite ESCRT-III polymers drives archaeal cell division

**DOI:** 10.1126/sciadv.ade5224

**Published:** 2023-03-15

**Authors:** Fredrik Hurtig, Thomas C. Q. Burgers, Alice Cezanne, Xiuyun Jiang, Frank N. Mol, Jovan Traparić, Andre Arashiro Pulschen, Tim Nierhaus, Gabriel Tarrason-Risa, Lena Harker-Kirschneck, Jan Löwe, Anđela Šarić, Rifka Vlijm, Buzz Baum

**Affiliations:** ^1^ Medical Research Council Laboratory of Molecular Biology, Cambridge CB2 0QH, UK.; ^2^ Molecular Biophysics, Zernike Institute for Advanced Materials, University of Groningen, 9747 AG Groningen, Netherlands.; ^3^ Laboratory of Soft Matter Physics, The Institute of Physics, Chinese Academy of Sciences, Beijing, China.; ^4^ University College London, Institute for the Physics of Living Systems, WC1E 6BT London, UK.; ^5^ Institute of Science and Technology Austria, 3400 Klosterneuburg, Austria.

## Abstract

ESCRT-III family proteins form composite polymers that deform and cut membrane tubes in the context of a wide range of cell biological processes across the tree of life. In reconstituted systems, sequential changes in the composition of ESCRT-III polymers induced by the AAA–adenosine triphosphatase Vps4 have been shown to remodel membranes. However, it is not known how composite ESCRT-III polymers are organized and remodeled in space and time in a cellular context. Taking advantage of the relative simplicity of the ESCRT-III–dependent division system in *Sulfolobus acidocaldarius*, one of the closest experimentally tractable prokaryotic relatives of eukaryotes, we use super-resolution microscopy, electron microscopy, and computational modeling to show how CdvB/CdvB1/CdvB2 proteins form a precisely patterned composite ESCRT-III division ring, which undergoes stepwise Vps4-dependent disassembly and contracts to cut cells into two. These observations lead us to suggest sequential changes in a patterned composite polymer as a general mechanism of ESCRT-III–dependent membrane remodeling.

## Teaser

*S. acidocaldarius*, an archaeon, divides via the patterned assembly and step-wise disassembly of a composite ESCRT-III polymer.

## INTRODUCTION

ESCRT-III proteins form polymers that play a critical role in membrane remodeling across the tree of life. In bacteria, ESCRT-III proteins, variably termed PspA/Vipp1/IMO30, have been shown to function in membrane repair and biogenesis (*1*, *2*). In archaea and eukaryotes, ESCRT-III proteins form polymers that function together with the AAA–adenosine triphosphatase (ATPase) Vps4 to cut membrane tubes from the inside to execute a wide variety of biological processes from membrane repair to compartment sealing, vesicle formation, and abscission (*3*–*6*). Although the precise mechanism by which ESCRT-III proteins and Vps4 remodel and cut membranes remains an unresolved question in the field, purified ESCRT-III proteins have been shown to form composite polymers that assume a wide variety of architectures, from flat spirals to cones and helices (*7*–*12*). In addition, reconstitution experiments have shown that ESCRT-III copolymers can undergo stepwise changes in their composition in the presence of adenosine 5′-triphosphate (ATP) and Vps4, which can drive changes in membrane curvature that lead to membrane tube formation and ultimately membrane scission (*13*–*17*). Furthermore, physical computational models of the process show that sequential changes in polymer structure are likely sufficient to drive membrane remodeling and scission (*18*, *19*). In addition, a recent theoretical analysis (*18*) has suggested that differences in the structure of different ESCRT-III polymers and in their affinity for membranes, when aided by the disassemblase activity of the AAA-ATPase, Vps4, are sufficient to generate the ordered changes in copolymer composition required for membrane deformation.

Currently, however, there is little evidence for stepwise changes in polymer organization driving membrane remodeling and scission in vivo. An exception to this is the case of cell division in *S. acidocaldarius*, one of the closest experimentally tractable archaeal relatives of eukaryotes (*20*, *21*). In these archaea, as is the case for several other Thaumarchaeota, Aigarchaeota, Crenarchaeota, Korarchaeota (TACK), and Asgard archaea (*22*–*25*), cell division appears to depend on a set of ESCRT-III proteins working together with Vps4 (also termed CdvC) (*24*, *26*). Because the ~1.25-μm diameter *S. acidocaldarius* division rings assemble and constrict with precise timing during the cell cycle, these cells provide an excellent model system to dissect the events involved in ESCRT-III/Vps4-dependent membrane remodeling. Using this system, we recently showed that proteasomal degradation of CdvB, one of the three main ESCRT-III proteins, triggers the transition from medial ring assembly to cytokinetic ring constriction and abscission (*27*). However, in this previous study technical limitations prevented determination of the precise subcellular organization of the different ESCRT-III polymers. Here, using a combination of ~30-nm resolution stimulated emission depletion (STED) microscopy, live imaging, and computational modeling, we have been able to determine the subcellular organization of the ESCRT-III polymers during division and to explore the role of Vps4 in this process. By studying division in control cells and in cells expressing a dominant-negative version of Vps4, we show that: (i) CdvB, CdvB1, and CdvB2 polymers exhibit distinct behaviors and have different preferred curvatures; (ii) CdvB, CdvB1, and CdvB2 assemble at spatially distinct positions relative to one another at the cell midzone to generate a prepatterned composite division ring; (iii) CdvB1 and CdvB2 polymers maintain their relative positions as rings constrict; and (iv) membrane scission depends on disassembly of polymeric CdvB2 from the cytokinetic bridge. Together, these data lead us to propose a framework for ESCRT-III/Vps4 function during archaeal cell division that we believe can be usefully applied to other instances of ESCRT-III–dependent membrane remodeling in both archaea and eukaryotes.

## RESULTS

**Fig. 1.**
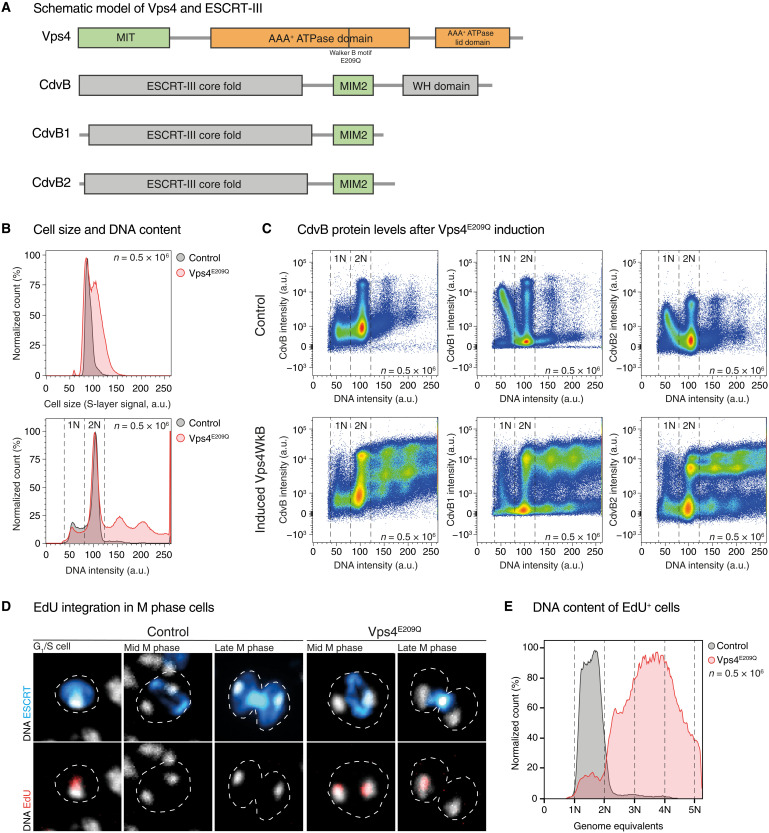
Induction of Vps4^E209Q^ causes cell division arrest but not cell cycle arrest. (**A**) Schematic model of Vps4 and ESCRT-III proteins in *S. acidocaldarius*, in which the approximate location of the Walker B point mutation, E209Q, in Vps4 has been marked. "WH" indicates winged helix domain. (**B**) Flow cytograms of control MW001 cells and Vps4^E209Q^ cells induced for 8 hours, showing a Vps4^E209Q^-induced increase in cell size (top) and in the 2N^+^ cell population (bottom). The DNA signal was visualized using 4′,6-diamidino-2-phenylindole (DAPI). The S-layer signal in arbitrary units was used as a proxy for cell size. (**C**) Flow cytograms of control MW001 cells and Vps4^E209Q^ cells were induced for 8 hours with staining against CdvB, CdvB1, and CdvB2, showing the accumulation of ESCRT-III proteins in Vps4^E209Q^-induced samples. (**D**) Control and STK + Vps4^E209Q^ cells at 4 hours post-Vps4 induction. (**E**) Flow cytometric analysis of cells stained with EdU conjugated to Alexa Fluor 647, showing the population of cells positive for EdU, indicative of DNA synthesis.

To investigate the role of Vps4 in the regulation of ESCRT-III–mediated cell division in *S. acidocaldarius*, we generated an arabinose-inducible expression plasmid carrying Vps4 with a E209Q point mutation in the Walker B motif (Fig. 1A), hereafter called Vps4^E209Q^. This point mutation prevents AAA-ATPases like Vps4 from hydrolyzing bound ATP and can therefore interfere with the activity of endogenous Vps4 complexes when overexpressed (*24*, *28*). The induction of Vps4^E209Q^ in *S. acidocaldarius* led to a notable increase in the population of large cells with a DNA content of greater than 2N (Fig. 1B and fig. S1A), as previously reported (*24*), and to an increase in the percentage of cells containing elevated levels of the ESCRT-III proteins, CdvB, CdvB1, and CdvB2 (fig. S1B), as well as Vps4^E209Q^ (fig. S1C). In these cultures, the majority of cells with a >2N DNA content expressed high levels of CdvB, CdvB1 and CdvB2 (Fig. 1C). At the same time, we observed a corresponding loss of newly divided G_1_ cells (fig. S1D), identified as a population of cells with a 1N DNA content and high levels of CdvB1 and CdvB2. This is consistent with the hypothesis that the expression of Vps4^E209Q^ prevents ESCRT-III polymer disassembly and cell division. While a similar pattern of ESCRT-III protein accumulation and division arrest has been reported to occur following the treatment of cells with a proteasomal inhibitor (*27*), the impact of the two treatments on cell cycle progression is different. Cells treated with the proteasomal inhibitor Bortezomib are both unable to divide and unable to initiate the next round of DNA replication (*27*), whereas cells prevented from dividing as a result of Vps4^E209Q^ expression undergo multiple additional rounds of DNA replication, leading to the steady accumulation of cells with an ever-larger DNA content (fig. S1E). As a further test of this finding, we expressed the Vps4^E209Q^ plasmid in a cell line carrying a thymidine kinase homolog (hereafter called STK). This makes it possible to visualize new DNA synthesis through the incorporation of the thymidine analog EdU by fluorescence microscopy (*29*). In control cells, EdU-labeled DNA was never observed in cells undergoing DNA segregation or cell division. Instead, EdU was only incorporated into the DNA of small newly divided cells (identified by the presence of high levels of cytoplasmic CdvB1 content), as expected based on the fact that wild-type cells initiate DNA replication shortly after entering G_1_ (Fig. 1D) (*27*, *30*). By contrast, when cell division was inhibited by the expression of Vps4^E209Q^, EdU incorporation was detected in dumbbell-shaped cells and in fully constricted cells connected by thin ESCRT-III–positive cytokinetic bridges. In many of these cells, EdU was seen in only one of the two separated nucleoids, indicating that the connecting bridge is not enough to ensure coordinated origin firing in the two daughter cells. Similar results were seen when EdU was visualized using flow cytometry. In control STK-positive cells, EdU accumulated in cells with <2N DNA content. By contrast, high levels of EdU were observed in cells expressing Vps4^E209Q^ with genome equivalents ranging from 1 to >5, with a peak at ~3.5 genome equivalents (Fig. 1E and fig. S1F). Thus, in stark contrast to the behavior of cells treated with proteasome inhibitor, Vps4^E209Q^ expressing cells continue to undergo repeated rounds of DNA replication following division arrest. In this, *Sulfolobus* cells resemble typical eukaryotes in which origin relicensing at the end of each cell division cycle requires the proteasome but not cytokinesis (*31*–*33*).

**Fig. 2.**
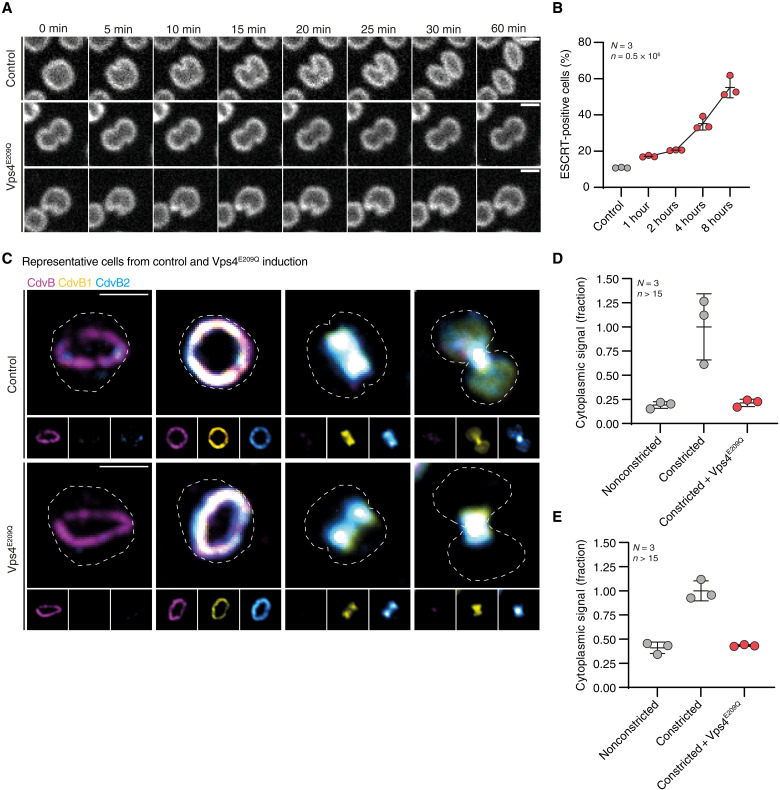
Vps4 is required for cell division. (**A**) Montage of images taken from live imaging of MW001 control and Vps4^E209Q^ after 2 hours of induction labeled with CellMask Plasma Membrane Stain. Scale bars, 1 μm. (**B**) Quantified flow cytometry data showing cells positive for CdvB or CdvB2 in MW001 control or in induced Vps4^E209Q^. Data exclude cells with <2N content. (**C**) Representative immunofluorescence microscopy images of MW001 control and Vps4^E209Q^ after 2 hours of induction; larger image is a color composite of the smaller images. Scale bars, 1 μm. Outline of cell shape deduced from S-layer staining. Data from three biological replicates (*N* = 3). (**D**) Quantification of cytoplasmic signal intensity for CdvB1 in MW001 control and Vps4^E209Q^ cells after 2 hours of induction. (**E**) Quantification of CdvB2 cytoplasmic signal intensity in MW001 control and Vps4^E209Q^ cells after 2 hours of induction.

To verify that Vps4 activity is required for ESCRT-III–mediated cell division, as suggested by this analysis, we upgraded our Sulfoscope (*34*) through the addition of a SoRa spinning disk and imaged divisions live in control cells and in cells expressing Vps4^E209Q^ that had been prelabeled with CellMask Plasma Membrane Stain (see Materials and Methods). As previously published (*35*), the rate of cytokinesis (diameter/unit time) in control MW001 cells was roughly constant once cell division had been initiated. By contrast, a short induction of Vps4^E209Q^ expression (2 hours) was sufficient to arrest cells at various points in the division process. In almost all cases, the expression of Vps4^E209Q^ prevented both ongoing midzone constriction and abscission (Fig. 2A and fig. S2A). Given the well-established role of Vps4 in disassembling ESCRT-III polymers (*26*, *36*–*38*), it seemed likely that this phenotype resulted from an inability of cells to remodel ESCRT-III division rings. In line with this, flow cytometry analysis revealed a substantial increase in the percentage of cells positive for CdvB, CdvB1, or CdvB2: from 10% in control cells to 55% after 8 hours of Vps4^E209Q^ induction (Figs. 1C and 2B and fig. S2, B to D). When imaged using immunofluorescence in fixed samples, Vps4^E209Q^ expressing cells were associated with an accumulation of division rings at all stages in the division process (Fig. 2C). The constriction of CdvB1 and CdvB2 rings in control cells was associated with a >5-fold increase in cytoplasmic signal for CdvB1 and a 2.5-fold increase for CdvB2 (Fig. 2, D and E), indicative of polymer disassembly during cytokinesis (*27*). However, following Vps4^E209Q^ expression, the entire pool of CdvB1 and CdvB2 localized to the ring even at late stages of ring constriction with no apparent cytoplasmic pool (Fig. 2, D and E). This analysis revealed another clear distinction between the effects of Vps4^E209Q^ expression and proteasomal inhibition. While cells treated with Bortezomib are unable to degrade CdvB and arrest with large diameter division rings containing high levels of CdvB, CdvB1, and CdvB2 (*27*), the expression of Vps4^E209Q^ blocked rings at every stage of constriction (Fig. 2C). Note, however, that neither Bortezomib treatment nor Vps4^E209Q^ appears to impair the ability of cells to assemble CdvB, CdvB1, and CdvB2 rings.

**Fig. 3.**
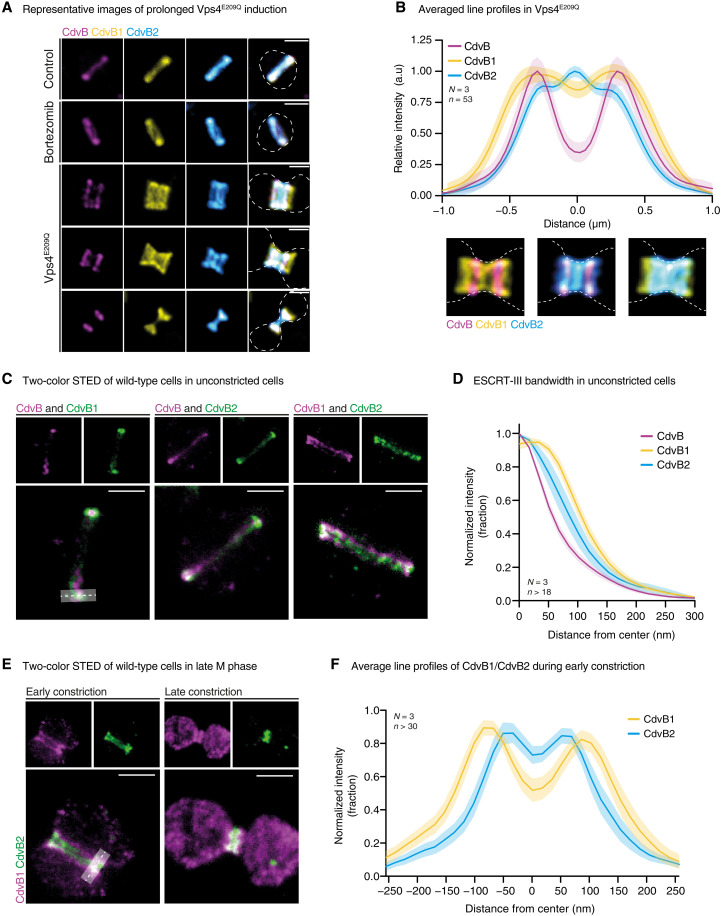
CdvB, CdvB1, and CdvB2 have different relative localization and roles. (**A**) Montage shows prevalent cell morphologies following 8 hours of Vps4^E209Q^ overexpression. Outline of cell shape was deduced from S-layer staining. Scale bars, 1 μm (*N* = 3). (**B**) Quantification of preferred ESCRT-III homolog localization from images like those presented in (A). Data collection used a 2 μm by 25 pixel line profile and was limited to constricted cells expressing all three proteins. Outline of cell shape was defined using S-layer staining. (**C**) STED microscopy of unconstricted DSM639 wild-type cells. Scale bars, 0.5 μm (*N* = 3). Dashed line and shaded region indicate how data for (D) was collected. Figure is presented in two colors for clarity of visualization. (**D**) Line profile quantification of band width from images like those presented in (C). Data collection used a 600 nm by 15 pixel line profile and was limited to constricted cells expressing all three proteins. (**E**) STED microscopy of constricted DSM639 wild-type cells. Scale bars, 0.5 μm (*N* = 3). Figure is presented in two colors for clarity of visualization. (**F**) Line profile quantification of band structure from images like those presented in (E). Data collection used a 600 nm by 15 pixel line profile and was limited to cells at early stages of constriction.

Over longer periods of time, the expression of Vps4^E209Q^ resulted in even more pronounced division phenotypes, leading to the accumulation of large dumbbell-shaped cells in which the different ESCRT-III proteins occupied a distinct and relatively reproducible position. In these cells, CdvB formed well-defined rings on either side of the cytokinetic bridge but tended to be excluded from the widest part of the dumbbell and from the narrow portion of the cytokinetic bridge (Fig. 3A). CdvB2 was seen coating the membrane bridge connecting the two daughter cells. In addition, a subpool of CdvB2 colocalized with the CdvB ring, as is observed in dividing control cells before the onset of CdvB degradation. By contrast, CdvB1 appeared to have a much less well-defined position along the bridge and was seen accumulating in a relatively uniform manner across the entire neck of the dumbbell, extending out from the constricted CdvB2-rich region of the neck beyond the CdvB ring (Fig. 3A). This same pattern emerged when we averaged the levels of CdvB, CdvB1, and CdvB2 across the cytokinetic bridge of more than 50 cells arrested as dumbbells (Fig. 3B). Again, CdvB localized to the ends of the neck, CdvB2 was preferentially localized to midzone, and CdvB1 spanned the entire length of the bridge. When these cells were imaged using electron cryotomography, we could observe an electron-dense layer lining the membrane, extending out from the cytokinetic bridge (fig. S3A and movie S1). The extent and localization of this electron-dense layer correlated well with observed ESCRT-III and Vps4 localizations (Fig. 3A and fig. S2D) and has previously been suggested to be composed of ESCRT-III polymers (*39*).

When we repeated the Vps4^E209Q^ expression in ΔCdvB1 cells (*34*), CdvB and CdvB2 polymers no longer appeared spatially separated (fig. S3B). This implies that CdvB1 is required to facilitate the spatial separation of CdvB and CdvB2 polymers in this context. Furthermore, ΔCdvB1 cells expressing Vps4^E209Q^ tended to have a single CdvB ring rather than two, suggesting that CdvB1 may be required to nucleate a second CdvB ring, as cells expressing Vps4^E209Q^ continue to grow and cycle without dividing. Note that we were unable to generate ΔCdvB2 cells carrying the Vps4^E209Q^ plasmid. The formation of patterned distributions of ESCRT-III proteins over prolonged periods of Vps4^E209Q^ expression suggested that different ESCRT-III polymers take up distinct preferred positions in the division ring.

Because confocal microscopy limits our ability to observe similar patterns in wild-type cells, we used multicolor STED microscopy (see Materials and Methods) on fixed cells to increase the spatial resolution of our analysis to ~30 nm. Notably, when visualized by STED, CdvB, CdvB1, and CdvB2 exhibited clear differences in the patterns of accumulation at early stages of division. CdvB was found to localize into a single narrow ring positioned at the center of control cells (Fig. 3C). Furthermore, CdvB was flanked by two wider accumulations of CdvB1 and CdvB2, which could often be resolved into two proximal CdvB2 and two distal CdvB1 rings (Fig. 3, C and D, and fig. S3C). When these rings were observed face on, the diameter of the large CdvB division ring was found to be ~40 nm smaller than the diameters of both CdvB1 and CdvB2 rings (fig. S3D), implying that CdvB1 and CdvB2 rings may form tighter contacts with the membrane than CdvB.

We next examined cells that had begun to constrict following the loss of CdvB. At the early stages of the constriction process, CdvB2 could be seen localizing to a pair of bands in the middle of the cytokinetic furrow flanked by two distal bands of CdvB1 (Fig. 3, E and F). At late stages in the division process, when the wild-type cytokinetic furrow had constricted to a narrow neck, CdvB2 was present in a single linear structure that occupied the central portion of the bridge, whereas CdvB1 was seen throughout the cytoplasm likely as a result of polymer disassembly (Fig. 3E) (*27*). Together, these data suggest a clear order of Vps4-dependent ESCRT-III disassembly from composite division rings: beginning with the removal of CdvB, followed by the disassembly of CdvB1, and then CdvB2.

**Fig. 4.**
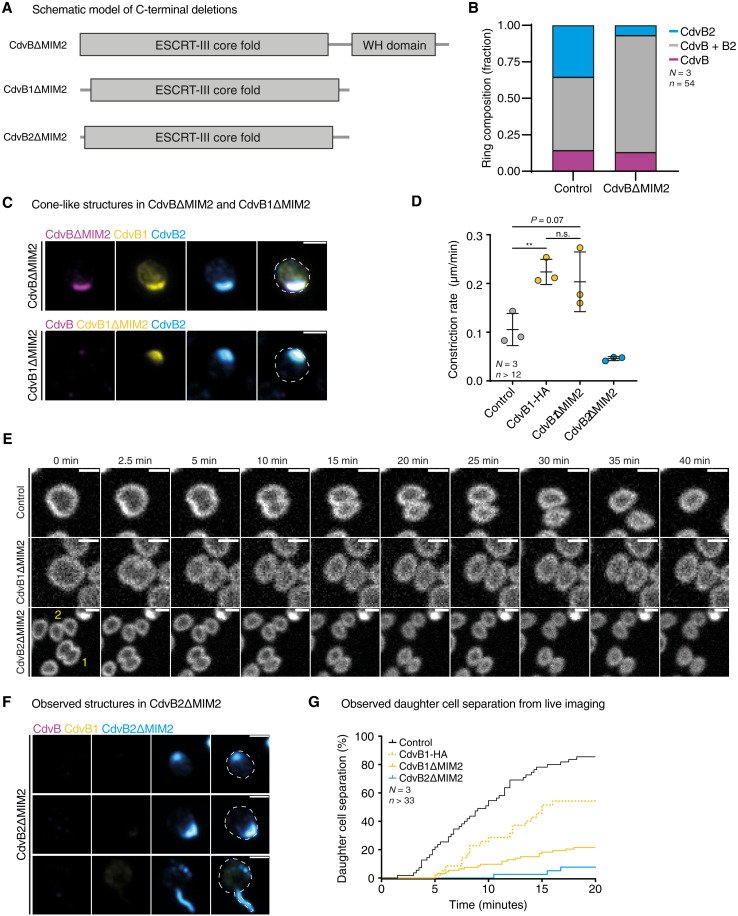
Sequential remodeling of CdvB1 and CdvB2 is required for proper constriction and cell division. (**A**) Schematic of ESCRT-III proteins constructs with MIM2 domain deletions. (**B**) Quantification of microscopy data showing the composition of division rings in MW001 control and CdvBΔMIM2 cells. (**C**) Montages of fluorescence imaging of CdvBΔMIM2 and CdvB1ΔMIM2 expressing cells. Scale bars, 1 μm. (**D**) Quantification of constriction speed from live imaging. Data were analyzed using a Mann-Whitney *U* test (**P = 0.0081; n.s., no significance). (**E**) Montages from live imaging of control as well as CdvB1ΔMIM2 and CdvB2ΔMIM2 expressing cells. Scale bars, 1 μm. (**F**) Immunofluorescence microscopy of CdvB2ΔMIM2 expressing cells showing localized accumulations and spike-like protrusions containing CdvB2ΔMIM2 protein. Scale bars, 1 μm. (**G**) Cumulative plot showing the percentage of daughter cells that have physically separatedwithin 20 min of completed constriction, as observed by live imaging of control, CdvB1, as well as CdvB1ΔMIM2 and CdvB2ΔMIM2 expressing cells.

In all systems studied thus far, Vps4 has been shown to be recruited to ESCRT-III polymers via the binding of its MIT domain to C-terminal MIT-interaction motif (MIM) domains on ESCRT-III proteins as a prelude to polymer disassembly (*6*, *24*, *40*, *41*). To determine how the Vps4-dependent disassembly of each of the three ESCRT-III polymers contributes to division in *S. acidocaldarius* cells, mutant versions of ESCRT-III proteins that lack the corresponding MIM2 domain (Fig. 4A) were overexpressed in an attempt to interfere with the stepwise disassembly described above (fig. S4A). The overexpression of CdvBΔMIM2 led to an accumulation of cells with wide, nonconstricted rings containing all ESCRT-III ring proteins, similar to the effect of Bortezomib treatment. This is consistent with the notion that MIM2-dependent removal of CdvB from the ESCRT-III copolymer ring by Vps4 is a prerequisite for its subsequent proteasomal degradation as well as constriction of the associated CdvB1 and CdvB2 rings (Fig. 4B). Overexpressed CdvB1ΔMIM2 was found to accumulate in rings (fig. S4B), like CdvBΔMIM2, but was more frequently observed in localized accumulations (Fig. 4C). However, CdvB1ΔMIM2 did not appear to interfere with constriction itself. Instead, CdvB1ΔMIM2 expression accelerated the rate of constriction measured by live imaging. While control cells constricted at an average rate of 0.11 μm/min (SD = 0.033), CdvB1ΔMIM2 cells displayed an average constriction rate of 0.20 μm/min (SD = 0.061) (Fig. 4, D and E). Overexpression of the full-length CdvB1 led to a similar increase in constriction speed (0.22 μm/min, SD = 0.026) (Fig. 4D), indicating that the observed increase in constriction speed was not due to loss of the MIM2 region. By contrast, cells lacking CdvB1 were previously shown to take longer to complete division (*34*). Notably, CdvB2ΔMIM2 expression had a very different impact on cell division from CdvB1ΔMIM2 expression. Cells with high levels of CdvB2ΔMIM2 frequently exhibited rigid (movie S2) spike-like protrusions (Fig. 4F and fig. S4C) along with conical bulges (fig. S4C) and, very occasionally, CdvB2ΔMIM2-rich cytokinetic bridges (fig. S4D). While the expression of CdvBΔMIM2, CdvB1ΔMIM2, and CdvB2ΔMIM2 all led to the accumulation of stable structures, the set of ESCRT-III proteins accumulating in each these structures followed a relatively strict rule (fig. S4E). CdvBΔMIM2-positive structures contained both CdvB1 and CdvB2, while the conical structures forming in CdvB1ΔMIM2 expressing cells contained CdvB2 but not CdvB. Last, CdvB2ΔMIM2-positive structures did not contain either of the other proteins (fig. S4E). These data support a sequential model of ESCRT-III polymer disassembly: CdvB is disassembled first, followed by CdvB1, and then CdvB2.

While the initial rates of constriction in CdvB2ΔMIM2 cells were slightly reduced as compared to the control (Fig. 4D and fig. S4F), CdvB2ΔMIM2 daughter cells were only very rarely observed to undergo successful abscission (Fig. 4G). Thus, while 85.5% of control daughter cells separated within 20 min of the end of constriction, less than 8% of CdvB2ΔMIM2 daughter cells separated within this time frame. This delay in abscission was observed to a much lesser extent in CdvB1ΔMIM2 expressing cells (21.5%) and was largely absent following the overexpression of full-length CdvB1 (54.3%) (Fig. 4G). These data support the idea that CdvB2 polymers: (i) have a preferred curvature compatible with their accumulation in very thin membrane tubes (internal diameter ≈ 75 nm), (ii) stabilize membrane tubes, and (iii) must be disassembled for abscission to occur. These observations also suggest that a portion of the thin CdvB2-rich spikes are likely to be remnants of CdvB2ΔMIM2 bridges that broke during processing for microscopy. Extracellular vesicle formation in *S. acidocaldarius* was also found to be depend on CdvB2, but not CdvB1 (fig. S4G), in line with CdvB2 playing a crucial role in membrane scission.

**Fig. 5.**
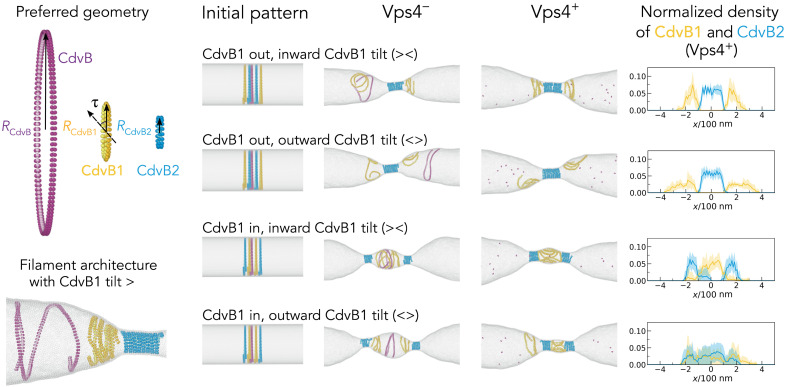
Simulations of membrane constriction in the presence and absence of active Vps4. Impact of the recruitment pattern of CdvB1 and CdvB2 on filament constriction and spatial organization without Vps4 (Vps4^−^) and in the presence of Vps4 (Vps4^+^). Left hand panels: The filaments are modeled as short helical strands with 1.05 loops in length, the initial radii equal to the cell radius, and preferred geometries as shown on the top: *R*_CdvB_ = 170 nm, *R*_CdvB1_ = 65 nm, *R*_CdvB2_ = 40 nm, CdvB1 tilt τ_𝐶𝑑𝑣𝐵1_ = 40^∘^, and filament bond stiffness *k*_CdvB_ = *k*_CdvB2_ = 250 *k*_B_*T*/σ^2^, *k*_CdvB1_ = 50 *k*_B_*T*/σ^2^. *T* is temperature, *k*_B_ is the Boltzmann constant, and σ is the simulation unit of length. The membrane is depicted in light gray. Bottom panel: the equilibrated geometry when CdvB1 is given an inward tilt of a magnitude τ_𝐶𝑑𝑣𝐵1_ = 40^∘^. Central panels: Initial conditions were defined such that CdvB (purple) forms a single ESCRT-III polymer at the cell center, which is flanked by CdvB1 (yellow) and CdvB2 (cyan) polymers. Simulation snapshots were then taken as the system was allowed to reach equilibrium as filaments tended toward their preferred curvature. Right: The normalized filament density of CdvB1 and CdvB2 after disassembly of CdvB (Vps4^+^) was calculated on the basis of data averaged more than 10 independent simulations, with SDs indicated by the shaded areas.

Together, these results imply functionally distinct roles for CdvB, CdvB1, and CdvB2, as the three proteins form homopolymers with distinct localizations and preferential curvatures—something that became evident when these proteins were imaged in cells several hours after the induction of Vps4^E209Q^ expression (Fig. 3A). To test the functional significance of this patterned assembly of ESCRT-III polymers, we used coarse-grained molecular dynamics simulations (*19*, *35*). In this expanded computational model, three ESCRT-III polymers representing CdvB, CdvB1, and CdvB2 were preassembled together on the membrane near the center of a tube designed to represent the cell in its predivision state. Polymers were then allowed to equilibrate and reach their mechanical equilibrium conformations. Because CdvB formed single well-defined rings in nonconstricted cells (Fig. 3, C and D), CdvB was modeled as a single polymer that preferentially forms rings with a large radius that is equal to the inner radius of the membrane. By contrast, CdvB2 rings were modeled as helical polymers with a smaller radius, in line with observations in wild-type, Vps4^E209Q^ and CdvBΔMIM2 cells (Figs. 3C and 4F and fig. S4D). Under these assumptions, the parameter space was scanned to determine a set of mechanical properties of CdvB1 that would enable the three polymers to physically separate in simulations (Fig. 5A). The spatial separation of polymers occurred more readily when CdvB1 filaments were modeled as being relatively flexible compared to CdvB and CdvB2, with a tilt of the membrane binding interface relative to the helical polymer axis and/or an intermediate preferred curvature between that of CdvB and CdvB2 (fig. S5, A to D). The tilt, as defined in (*19*), determines the orientation of the polymer’s membrane attraction site relative to its helical axis. When these three properties were combined in simulations, flexible CdvB1 polymers with a tilt of 40° were seen to create cone-shaped membranes and the necks of dumbbell-shaped cells, consistent with the pattern of CdvB1 localization observed in Vps4^E209Q^ expressing cells (Fig. 3A). Using these parameters, we then tested the importance of precisely positioning the different ESCRT-III polymers on the membrane before division, in the presence or absence of Vps4 activity. In these simulations, reproducible formation of a single constriction site required the assembly of two CdvB1 rings placed outside of the two central CdvB2 rings before the onset of cytokinesis (Fig. 5; fig. S6A; and movie S3, A and B), implying a role for patterned assembly in ESCRT-III–dependent division.

## DISCUSSION

Together, these findings demonstrate an important role for the ordered and prepatterned assembly of ESCRT-III filaments and their subsequent Vps4-dependent sequential disassembly for cell division in *S. acidocaldarius*. They also suggest a sequential model for ESCRT-III–dependent cell division. This begins with the assembly of a CdvB ring at the center of cells that are ready to divide. This single CdvB ring acts as a noncontractile template upon which CdvB1 and CdvB2 can polymerize in a manner that does not depend on Vps4 (*13*, *42*).

STED microscopy revealed that CdvB, CdvB1, and CdvB2 formed spatially separated rings in wild-type cells before the onset of constriction (Fig. 3, C and D, and fig. S3D). This suggests that ESCRT-III proteins in *S. acidocaldarius* form layered composite structures, not heteropolymers like their eukaryotic counterparts Vps2 and Vps24 (*43*). CdvB1 consistently formed two rings flanking CdvB2, while CdvB2 was found either assembled into a single diffuse structure overlying the central CdvB ring or into two flanking rings that are too close to easily resolve using STED. Then, following the onset of cytokinesis, both CdvB1 and CdvB2 signals resolved into two pairs of rings. These data imply that the spatial separation of ESCRT-III polymers may be accentuated as the membrane deforms; something that can also be observed in simulations as polymers seek out regions of suitable curvature (Fig. 5A). Although it is not clear from our analysis exactly how the placement of these different ESCRT-III rings is achieved, these simulations show that a similar spatial separation can be induced by simple differences in the structure and mechanics of the different homopolymers.

During ring constriction, CdvB1 and CdvB2 were found to retain their respective positions, with CdvB1 polymer extending outside of the two central CdvB2 rings. However, once constriction was near complete, CdvB was largely absent and CdvB1 was almost entirely cytoplasmic, leaving cells connected by a thin CdvB2-rich neck. These data suggest that while the majority of the CdvB pool is disassembled and degraded before the onset of ring constriction, as previously reported (*22*), the disassembly of CdvB1 begins early in the process of constriction. This is followed, with some delay, by the disassembly of CdvB2. CdvB2 disassembly is likely to be the event that produce scission in *S. acidocaldarius*, since cells expressing CdvB2ΔMIM2 form doublets connected with narrow bridges that fail to separate. These data are in line with data in *Saccharolobus islandicus* (*44*) and with simulations that suggest a role for disassembly in scission (*27*, *35*). The importance of CdvB2 for division was also clear from the profound division defect observed in *Sulfolobus* cells lacking the CdvB2 protein, implying that the function of CdvB2 is poorly rescued by the presence of CdvB1 (*34*). Furthermore, the deletion of CdvB2 completely abolished the generation of extracellular vesicles (fig. S4G), while CdvB1 was dispensable for vesicle formation, further supporting a critical role for CdvB2 in membrane scission. The contrast between CdvB1 and CdvB2 function was also clear in MIM2 deletion experiments in which the expression of CdvB1ΔMIM2 or CdvB1 was found to accelerate constriction, probably by increasing the cellular concentration of CdvB1. These data, together with the fact that the loss of CdvB1 leads to longer division times and occasional division failures (*34*), suggest that the flanking CdvB1 polymers facilitate CdvB2 positioning, robust constriction, and CdvB2-dependent scission, as suggested by simulations (Fig. 5A and fig. S6, A and B).

Having detailed the spatial organization of ESCRT-III polymers during cytokinesis in *Sulfolobus* cells, it is worth comparing this to what is known about ESCRT-III–dependent abscission in human cells. In the latter case, one or two conical ESCRT-III spirals extend from either side of a stable midbody, leading to constriction at sites some distance away from the midbody where abscission occurs (*14*, *45*–*47*). By contrast, in *Sulfolobus* cells, the constricting ESCRT-III polymers that drive division appear to take up a symmetrical organization in which two cones are oriented point to point (> <). In this way, the ESCRT-III rings in *Sulfolobus* define a single central site at which membrane remodeling and scission occurs. One of the key reasons for this difference between the archaeal and mammalian systems may be the loss of the CdvB template at the center of the bridge in *Sulfolobus* cells, which is occluded by the midzone in human cells.

As is the case in human cells, our analysis also shows that cell cycle progression in *Sulfolobus* is uncoupled from ring constriction. Thus, both Vps4^E209Q^ and CdvBΔMIM2 expressing cells arrest during division with large rings but undergo extensive endoreplication. This contrasts with the effect of proteasomal inhibition, which arrests both cell division and the cell cycle in *S. acidocaldarius*. This implies that while cell division progression requires proteasomal degradation of CdvB, the proteasome is also required for the degradation of an as of yet unidentified protein for origins of replication to be licensed and/or to fire in the following cycle.

In conclusion, this study shows how the patterned assembly and ordered Vps4-dependent disassembly of ESCRT-III copolymers is able to drive ESCRT-III–dependent membrane remodeling and membrane scission in a simple in vivo model of ESCRT-III/Vps4 function. In this way, our study supports and extends the conclusions of recent in vitro studies using the equivalent eukaryotic counterparts, which undergo Vps4-dependent changes in composition (*13*). In the future, it will be important to improve our understanding of the similarities and differences between the process described here and Vps4-dependent ESCRT-III–mediated membrane remodeling in eukaryotes.

## MATERIALS AND METHODS

### Cell culturing, fixing, and drug treatment

*S. acidocaldarius* DSM 639 (background strain), MW001 (uracil auxotrophic cloning strain), STK, and all plasmid carrying strains were grown in Brock medium (pH 2.9) supplemented with 0.1% N-Z-amine and 0.2% sucrose at 75°C. MW001 and STK was supplemented with uracil (4 μg/ml) where needed. Cells were fixed by stepwise addition of 4°C ethanol to a final concentration of 70% and then stored at 4°C. Proteasome inhibition was performed by treating cells with 10 μM bortezomib (Abcam, ab142123) for 40 min. STK strains were supplemented with 200 μM EdU (Thermo Fisher Scientific, C10639) for 10 min.

### Cloning, transformation, and overexpression

The Vps4^E209Q^ plasmid was created by amplifying Vps4 (Saci_1372) from *S. acidocaldarius* DSM 639 and generating a point mutation in the Walker B motif at glutamic acid 209 to glutamine and tagged with a 6xHis tag on the C terminus. CdvBΔMIM2 was generated by amplifying CdvB (Saci_1373) from *S. acidocaldarius* DSM 639 in two blocks, excluding the MIM2 domain (residues 183 to 193). CdvB1ΔMIM2 and CdvB2ΔMIM2 were generated by amplifying CdvB1 (Saci_0451) and CdvB2 (Saci_1416) from *S. acidocaldarius* DSM 639 up to the point of the MIM2 motif but no further (excluding residues 198 to 214 and 198 to 219, respectively). Fragments were cloned into the pSVAaraFX backbone for Vps4^E209Q^ and the pSVAaraHA-stop backbone for CdvBΔMIM2, CdvB1ΔMIM2, and CdvB2ΔMIM2 using Gibson Assembly [New England Biolabs (NEB), E5510S]. Plasmids were methylated in vivo before transformation into *S. acidocaldarius* by transformation into *Escherichia coli* ER1821 (NEB). Electrocompetent *S. acidocaldarius* MW001 were transformed using electroporation (2000 V, 25 μF, 600 ohms, 1 mm) and selected on Gelrite-Brock plates, following established practices in *Sulfolobus* genetics (*48*).

### Immunolabeling

Fixed cells were rehydrated in PBSTA (phosphate-buffered saline supplemented with 0.2% Tween 20 and 3% bovine serum albumin). Cells were then incubated overnight at 25°C and 500 revolutions per minute (rpm) agitation with PBSTA supplemented with 5% fetal bovine serum (FBS) and primary antibodies (table S3). Primary antibodies were detected using secondary antibodies (table S4) for 3 hours at 25°C and 500 rpm agitation. S-layer labeling for cell outlines was performed by incubating cells with Concanavalin A conjugated to Alexa Fluor 488 (Thermo Fisher Scientific, C11252) or Alexa Fluor 647 (Thermo Fisher Scientific, C21421) at 50 μg/ml during secondary antibody incubation. DNA was labeled by addition of 2 μM Hoechst (Thermo Fisher Scientific, 62249) or 3 μM DAPI (4′,6-diamidino-2-phenylindole; Thermo Fisher Scientific, 62248) after secondary antibody incubation. EdU labeling was performed by a Click-iT kit according to the manufacturer’s instructions (Thermo Fisher Scientific, C10640). For spinning disc microscopy, Lab-Tek chambered slides (Thermo Fisher Scientific, 177437PK) were coated with 2% polyethyleneimine (PEI) overnight at room temperature. Chambers were washed with Milli-Q water before stained cell suspension was added and spun down for 1 hour at 750 relative centrifugal force (RCF). For STED microscopy, 18-mm coverslips (borosilicate no. 1.5, Marienfeld, 0117580) were coated with 2% PEI overnight at room temperature before washing with Milli-Q water. Coverslips were placed in 24-well plates and spun down with labeled cell suspensions for 1 hour at 750 RCF. Coverslips were mounted on microscopy slides using 10 μl of homemade Mowiol mounting media and allowed to cure overnight at room temperature.

### Spinning disc microscopy

Cells were imaged in Lab-Tek chambered coverslip using a Nikon Eclipse Ti2 inverted microscope equipped with a Yokogawa SoRa scanner unit and Prime 95B scientific complementary metal-oxide semiconductor (sCMOS) camera (Photometrics). Images were acquired with a 100× oil immersion objective (Apo TIRF 100×/1.49, Nikon) using immersion oil (immersion oil type F2, Nikon). A total magnification of ×280 was achieved using the ×2.8 magnification lens in the SoRa unit. Images were acquired with 200-ms exposure time for labeled proteins and 500-ms exposure time for DNA stains, with laser power set to 20%. *z*-axis data were acquired using 10 captures with a 0.22-μm step. Analysis and *z*-axis maximum projections were performed using ImageJ and the ImageJ plugin ObjectJ (sils.fnwi.uva.nl/bcb/objectj/).

### STED microscopy

STED images were acquired of fixed samples using an Abberior Expert Line STED microscope with a 100× oil immersion objective (Olympus Objective UPlanSApo 100×/1.40 oil). Before imaging the confocal and STED, lasers were aligned using a 0.1-μm TetraSpeck (Invitrogen, T7279) bead sample. In addition, by switching secondary antibodies, we verified that none of the relative positions of the polymers were affected by the used fluorophores.

Fluorophores are subjected to photobleaching, limiting the cycles of excitation and deexcitation, before they transition into a nonfluorescent state. To minimize this photobleaching, adaptive illumination strategies RESCue (reduction of state transition cycles) and DyMIN (dynamic intensity minimum) were used (*39*, *40*). In RESCue mode, intensity thresholds are set at specific percentages of the dwell time. If these thresholds are not reached, then the lasers will be switched off for the remainder of the dwell time. As a result, imaging only occurs if a structure is present. In DyMIN mode, an intensity threshold was set on the confocal image, creating a low-resolution structure localization mask. A second more precise structure localization was determined by imaging within the masked region using a low-intensity STED beam. For the final image, a high-intensity STED beam was used to achieve the best attainable resolution.

All STED images were acquired with a pixel size of 17 nm and a 0.8AU pinhole. For excitation, we used 40-MHz pulsed lasers with a wavelength of 561 (200 μW at laser head) and 640 nm (1 mW at laser head). The STED depletion laser (also 40 MHz pulsed) has a head intensity of 3.2 W at 775 nm. We applied gated detection, with a window of 13 ns in the 640 channel and 14.3 ns in the 561 channel. The applied laser powers and dwell times (line steps multiplied with pixel dwell time) were optimized for the different samples as shown in table S1. For the detection of the emission into the 640 channel (640-nm laser excitation), the detection filter was set to 697/56 nm. For the detection of the 560 channel (561-nm laser excitation), the detection filter was set to 603/27 nm. For image acquisition, automated STED has been used as presented in (*49*). For image analysis, a script was written in Python 3 to quantify the relative protein ring positions, available at (*50*).

Control samples with secondary STED antibodies (table S2) on slides were prepared to estimate the achieved resolution. First, coverslips were coated with poly-l-lysine (PLL) for 2 hours and washed with Milli-Q water afterwards. A droplet of 10 μl of secondary antibody, Abberior STAR 580 (Abberior, ST580-1002-500ug, lot no. 90619CWF-3) or Abberior STAR 635 (Abberior, ST635-1002-500UG, lot no. 18052018-Hp), was pipetted on a piece of parafilm in a humid chamber. The PLL-coated coverslip was placed on top of the droplet and was incubated at room temperature for 1 hour. After incubation, 6 μl of homemade Mowiol was pipetted on a glass slide, and the coverslip with secondary antibody was put on top and left to dry overnight at room temperature, protected from light. For the imaging of these control samples, settings matching all STED measurements were used. For the analysis, ImageJ was used to draw line profiles and measure the full width at half maximum as a measure for the resolution, resulting in an estimated resolution of 29.6 ± 5.62 nm (*n* = 33, ±1 SD).

### Flow cytometry

DNA was labeled with 1.25 nM DAPI for flow cytometry. Flow cytometry analysis was performed on BD Biosciences LSRFortessa. Laser wavelengths and filters used were as follows: 355, 488, 561, and 633 nm, with filters 450/50 ultraviolet, 525/50 blue, 582/15 yellow-green, and 710/50 red, respectively. Side scatter and forward scatter was recorded. Analysis was performed using FlowJo v10.8.1.

### Western blotting

Cells were lysed in Laemmli buffer, followed by four cycles of sonication (30/30s on/off) at low power settings on a Bioruptor Plus sonication system (Diagenode, B01020001). Before running samples on a gel, samples were incubated at 99°C for 10 min. Ten microliters of samples were run on NuPAGE 4 to 12% Bis-Tris gels (Invitrogen) at 180 V using MES running buffer. Proteins were transferred to a nitrocellulose membrane and blocked with PBST + 5% milk for 1 hour, after which the membrane was stained with primary antibodies (table S3) in PBST + 5% milk and 5% FBS overnight at 4°C. The membrane was washed three times in PBST for 5 min and then incubated with PBST + 5% milk and secondary antibodies for 2 hours. The membrane was recorded using a Bio-Rad ChemiDoc system.

### Live imaging

Live-cell imaging was performed using the Sulfoscope microscopy setup as described in (*34*), with minor modifications. Briefly, 25-mm coverslips were washed with EtOH and H_2_O, then assembled into commercial Attofluor chambers, and covered with 300 μl of BNS. Chambers were incubated at 75°C until the media had dried, then washed thoroughly with BNS, and placed into the Sulfoscope to equilibrate to 75°C. For visualization of *S. acidocaldarius* membranes, cells at optical density at 600 nm (OD_600nm_) of 0.15 to 0.3 were supplemented with CellMask Deep Red Plasma Membrane Stain (0.75 μg/ml; Thermo Fisher Scientific) immediately before imaging. For imaging, 400 μl of cells were loaded into the Attofluor chamber and immobilized using heated, semi-solid BNS pads [0.6% Gelrite, 0.5× BNS (pH ~5), and a final concentration of 20 mM CaCl_2_]. Immobilization pads were prepared by cutting 7-mm length half-moon shapes from semi-solid BNS media (15 ml per plate), placed onto 13-mm circular coverslips, and incubated at 75°C for 5 to 10 min in a bead bath until the pads had slightly dried and the edges of the pad curved downwards. Preheated pads were then placed in the chamber such that the edge of the pad was in the center of the chamber. Cells found at the border of the immobilization pad were imaged, as these cells are only subject to reduced diffusion resulting in a moderate level of immobilization rather than directly in contact with the pad, limiting the mechanical stress they are under. Cells were allowed to settle for 5 to 10 min before acquisition on a Nikon Eclipse Ti2 inverted microscope equipped with a Yokogawa SoRa scanner unit and Prime 95B sCMOS camera (Photometrics). Images were acquired with a 60× oil immersion objective (Plan Apo 60×/1.45, Nikon) using a custom formulated immersion oil for high temperature imaging (maximum refractive index matching at 70°C, *n* = 1.515 ± 0.0005; Cargille Laboratories). A total magnification of ×168 was achieved using the ×2.8 magnification lens in the SoRa unit. Images were acquired with 15-ms exposure time and 10% laser power at intervals of 15 (one-color imaging) or 20 s (dual-color imaging) for 2 to 3 hours. After acquisition, the ImageJ plugin StackReg (*51*) was used to correct *XY* drift.

### Electron microscopy

Vps4^E209Q^ and CdvB2ΔMIM2 cells were induced for 8 hours and after pelleting and were resuspended in fresh medium at OD_600nm_ = 1.75 and 2.5, respectively. Three microliters of resuspended cells were applied to a freshly glow-discharged Quantifoil Au 200 mesh R2/2 or R2/1 grid. For tomography of Vps4^E209Q^ cells, 1 μl of 10-nm protein A–conjugated gold fiducials (BBI Solutions) was applied to the grid and blotted off from the back before cells were applied to the grid. Grids were blotted manually from the back inside a Vitrobot Mark III chamber (Thermo Fisher Scientific) set to 20°C and 100% humidity and plunge-frozen into liquid ethane maintained at −180°C using a cryostat (*52*). Grids were imaged at cryogenic temperature on a Glacios microscope (Thermo Fisher Scientific) equipped with a Falcon III detector and operating at 200 kV or a Titan Krios microscope (Thermo Fisher Scientific) equipped with a Quantum energy filter and K3 detector (both from Gatan) and operating at 300 kV. The presented micrographs were low- and high-pass filtered and contrast-adjusted in ImageJ 2.3 (*53*) for display purposes. Tomographic data were collected on a Titan Krios microscope (Thermo Fisher Scientific) operating at 300 kV and equipped with a Quantum energy filter and K2-XP detector (both from Gatan) at a pixel size of 4.44 Å, −5 to −8 μm defocus, and a total dose of 183 e^−^ Å^−2^ using a grouped dose-symmetric tilt scheme (−56° to +56°) (*54*) in SerialEM 3.9 (*55*). Data were processed in IMOD 4.11 (*56*) and Tomo3D 2.0 (*57*) using the SIRT reconstruction method. Reconstructed tomograms were low-pass filtered to 40 Å using EMAN 2.0.6 (*58*) for display purposes.

## Supplementary Materials

### This PDF file includes:

Supplementary Text Figs. S1 to S7 Tables S1 to S4 Legends for movies S1 to S3 References

## Notes

View/request a protocol for this paper from *Bio-protocol*.
