# Supplementary material for: The patterned assembly and stepwise Vps4-mediated disassembly of composite ESCRT-III polymers drives archaeal cell division
Source: Sci Adv. 2023 Mar 15;9(11):eade5224. doi: 10.1126/sciadv.ade5224 (PMC10017037; doi:10.1126/sciadv.ade5224)
Supplement: Supplementary file 1 — Supplementary Text Figs. S1 to S7 Tables S1 to S4 Legends for movies S1 to S3 References [file sciadv.ade5224_sm.pdf]

Science Advances
AAAS

Supplementary Materials for

The patterned assembly and stepwise Vps4-mediated disassembly of composite ESCRT-III polymers drives archaeal cell division

Fredrik Hurtig *et al.*

Corresponding author: Anđela Šarić, andela.saric@ist.ac.at; Rifka Vlijm, r.vlijm@rug.nl; Buzz Baum, bbaum@mrc-lmb.cam.ac.uk

Sci. Adv. **9**, eade5224 (2023)
DOI: 10.1126/sciadv.ade5224

The PDF file includes:

Supplementary Text
Figs. S1 to S7
Tables S1 to S4
Legends for movies S1 to S3
References

Other Supplementary Material for this manuscript includes the following:

Movies S1 to S3

Supplementary Text:

Computational details

The ESCRT-III filaments were initialized as five short polymers placed successively along the inner surface of a long, tubular, deformable membrane **(Fig. 5A)**. To model the filaments, we used the ESCRT-III model we developed in Harker-Kirschneck 2019 (*19*), in which the filament consists of three beaded monomers that are bonded to their neighbouring monomers via nine harmonic bonds. The bond strength determines the stiffness of the polymer, whereas the exact ratio of the bond lengths give the filament its chiral curvature. The filament can constrict or expand its target radius by modifying its bond lengths.

Here each filament consisted of 1.05 helical loops (82 monomers). Two filament recruitment patterns were investigated as the initial configuration of the molecular dynamics (MD) simulations: CdvB1-CdvB2-CdvB-CdvB2-CdvB1 (termed "CdvB1 out"), CdvB2-CdvB1-CdvB-CdvB1-CdvB2 (termed "CdvB1 in"). The target radii of CdvB (purple), CdvB2 (cyan), CdvB1 (yellow) were set as $R_{CdvB} = 17\sigma$, $R_{CdvB2} = 4.0\sigma$, $\frac{1}{R_{CdvB1}} = \left(\frac{1}{R_{CdvB}} + \frac{1}{R_{CdvB2}}\right)/2$ (i.e., $R_{CdvB1} = 6.5\sigma$), where σ is the MD unit of length and corresponds to roughly 10 nm. Filament bond stiffness was set as $k_{CdvB} = k_{CdvB2} = 250\ k_B T/\sigma^2$, $k_{CdvB1} = 50 k_B T/\sigma^2$. CdvB1 was given a tilt $\tau_{CdvB1} = 40°$, and we investigated whether the outward (< >) or inward (> <) conical direction at filament recruitment had an impact on the filament separation and constriction. The membrane was modelled with the one-particle-thick model developed by Yuan 2010 (*59*). The membrane tube consisted of 5000 beads and had an initial radius of $R_{tube} = 18\sigma$. The tube length in x direction along the simulation box was set to $l_{tube} = 360\ \sigma$. The size of the coarse-grained beads was $r_{membrane} = r_{subunit} = 0.5\ \sigma$. Short-ranged 12-6 Lennard-Jones (LJ) interactions were applied between the two bottom beads of the filament's three-beaded subunits and the membrane beads:

$$E_{ij} = 4\epsilon\left(\left(\sigma/r_{ij}\right)^{12} - \left(\sigma/r_{ij}\right)^{6}\right) - E_c\ , r_{ij} < r_{cut}.$$

The interaction strength was set to $\varepsilon_{LJ} = 3.0\ k_B T$ and the cut-off distance to $r_{cut} = 1.3 \cdot r_{min}$, where $r_{min} = 2^{\frac{1}{6}}\sigma$ is the inter-particle contact distance and E_c is the energy at cut-off distance. Volume exclusions was applied between different filament subunits and between the membrane and the top beads of the filament. The volume exclusion interactions were treated as LJ interactions truncated at r_{min} and shifted to zero, with the interaction strength set as to $\varepsilon_{volex} = 2.0\ k_B T$. Periodic boundary conditions were applied in three dimensions. We performed molecular dynamics simulations using the molecular dynamics package LAMMPS (*60*) and integrated the equations of motion with a timestep 0.01 t_0, where t_0 is the MD unit of time, coupled to a Langevin thermostat. The Langevin thermostat was applied at every step with the temperature set to 1 and the damping coefficient set to 1 t_0. The box size and the number of particles were initially kept constant. Initially the system was equilibrated for t=100t_0, with all three filament types having the same target radius R_{CdvB} = R_{CdvB1} = R_{CdvB2} = 17 σ. This allowed the membrane to attach to the filament loops from the outside. Then CdvB remained at R_{CdvB} = 17 σ, while CdvB1 and CdvB2 reduced their target radius to R_{CdvB1} = 6.5 σ and CdvB2 to R_{CdvB2} = 4.0 σ respectively. We then let the system to evolve for t = 2×10^5 t_0, until the formation of a stable filament distribution formed. In Vps4+ simulations, we disassembled the CdvB filament by severing its internal bonds and turned the interaction between bottom beads and membrane beads from attractive interactions to pure volume exclusion immediately after the initial equilibration. We carried out ten independent simulations for each setup and the configurations of the last snapshots of the trajectories were used to calculate the normalized filament density **(Fig. 5A, S6A-S6B)**. We visualize our simulation results using OVITO (http://ovito.org).

To see if this newly found filament alignment (CdvB1-CdvB2-CdvB1 with CdvB1 tilted inwards) can cause cells to divide, we placed two opposing CdvB1 filaments (each three loops long) around a single looped CdvB2 filament inside a membrane tube with R_{tube} = 27 σ. In this simulation filament curvature is changed progressively, by picking random monomers within the filament and constricting them, instead of releasing all the filament energy at once **(Fig.**

S7A). As shown in Harker-Kirschneck et al. 2022 with a single filament, this dynamical protocol led to the most reliable and symmetric division and came remarkably close to matching the kinetics of ring constriction measured from experimental data (*35*). In this new simulation setup with multiple ESCRT-III filaments, the cubical simulation box had a length of 400 σ and CdvB1 was given a large tilt $\tau_{CdvB1} = 90°$. The large tilt allowed CdvB1 to decrease the diameter of the membrane neck by providing a surface for the membrane to glide over and into the neck. The filament stiffness was set to $k_{CdvB1} = k_{CdvB2} = 400\, k_B T/\sigma^2$. After equilibrating the membrane on its own for 200 t_0, we started transitioning the CdvB1 filaments at random positions from the initial state (R_{CdvB1}= R_{tube}, τ_{CdvB1} = 0°) to its tilted and constricted final state (R_{CdvB1} = 60% R_{tube}, τ_{CdvB1} = 90°). Every 100 simulation steps one subunit in each CdvB1 filament got transformed, while the CdvB2 filament remained in its initial state (R_{CdvB2} = R_{tube}). Then the CdvB2 filament in the middle was transitioned by transforming one random subunit within the filament from a large to a small target radius every 10000 steps, until the entire filament was transitioned. This transition was much slower, as the target radius of CdvB2 has to decrease dramatically from $R_{CdvB2} = R_{tube}$ to $R_{CdvB2} = 1.75\sigma$ – by about 93.5%. After constriction was completed, we disassembled the filaments by severing the bonds between random subunits within the filament – first CdvB1 and then CdvB2, which led to division. We found that scission only occurs reliably if i) the target radius is very small, $R_{target} \leq 2\sigma$, and ii) we use the ensemble that preserves the constant zero pressure along the axis of the simulation box, instead of preserving the volume of the box. This maintains the membrane under low tension by allowing the membrane area to shrink/increase as needed (by adjusting the simulation box size). This supports the finding of Lafaurie 2013, who show that membrane tubes under tension do not divide (*61*).

When simulating filament separation, we initially placed the filaments on the membrane as three interlaced strands, consisting of two full helical loops **(Fig. S5A)**. Each filament strand was modelled by our three-bead-monomer ESCRT-III model (*19*) and consisted of 156 monomers. The membrane tube consisted of 30000 beads. The initial radius of the membrane

tube was $R_{tube} = 18\sigma$, with length in x direction $l_{tube} = 216\sigma$. Initially the system was equilibrated for t = $100t_0$ with CdvB, CdvB1 and CdvB2 all at the same target radius $R_{CdvB} = R_{CdvB1} = R_{CdvB2} = 17\sigma$, to ensure that all three filaments attached to the interior of the membrane tube. We then activated CdvB1 and CdvB2 by resetting their target radii, and allowed the system to evolve until the filaments were fully separated (typically 10^5 to 10^6 t_0). Time step $dt = 0.01t_0$ was used for all MD simulations.

Characterization of filament separation time (Fig. S7)

Firstly, we computed the overlap function (OF) by integrating the overlap of the normalized bead distribution along the x-axis of the two filaments of interest. OF = 0 means fully separated and OF = 1 means fully overlapped. The OF was averaged over five independent simulations. The filament separation time is defined as the first time when the averaged OF is below a certain threshold value. The threshold value was computed from the average of the OF over the last 100 frames.

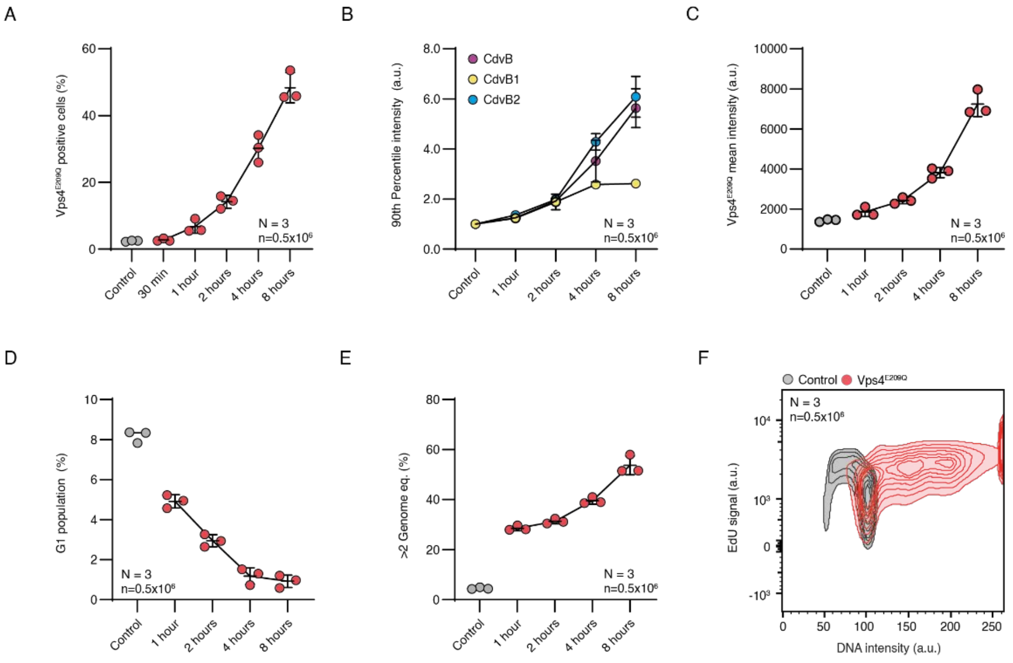

Figure S1. Induction of Vps4^{E209Q} causes cell division arrest but not cell cycle arrest. **(A)** Quantification of percent of population positive for Vps4^{E209Q} signal in flow cytometry. Limit for positive cells determined by control. **(B)** 90% percentile intensity of CdvB, CdvB1 and CdvB2 signal in flow cytometry. **(C)** Mean intensity of Vps4^{E209Q} signal in flow cytometry. **(D)** Quantification of G1 population size from flow cytometry. Gating was performed using a DNA vs CdvB2 cytogram. **(E)** Percent of cells with > 2 genome equivalents from flow cytometry in Vps4^{E209Q}. **(F)** 2D contour plot showing DNA intensity and EdU signal from flow cytometry. Contour cut off = 10% (N = 3).

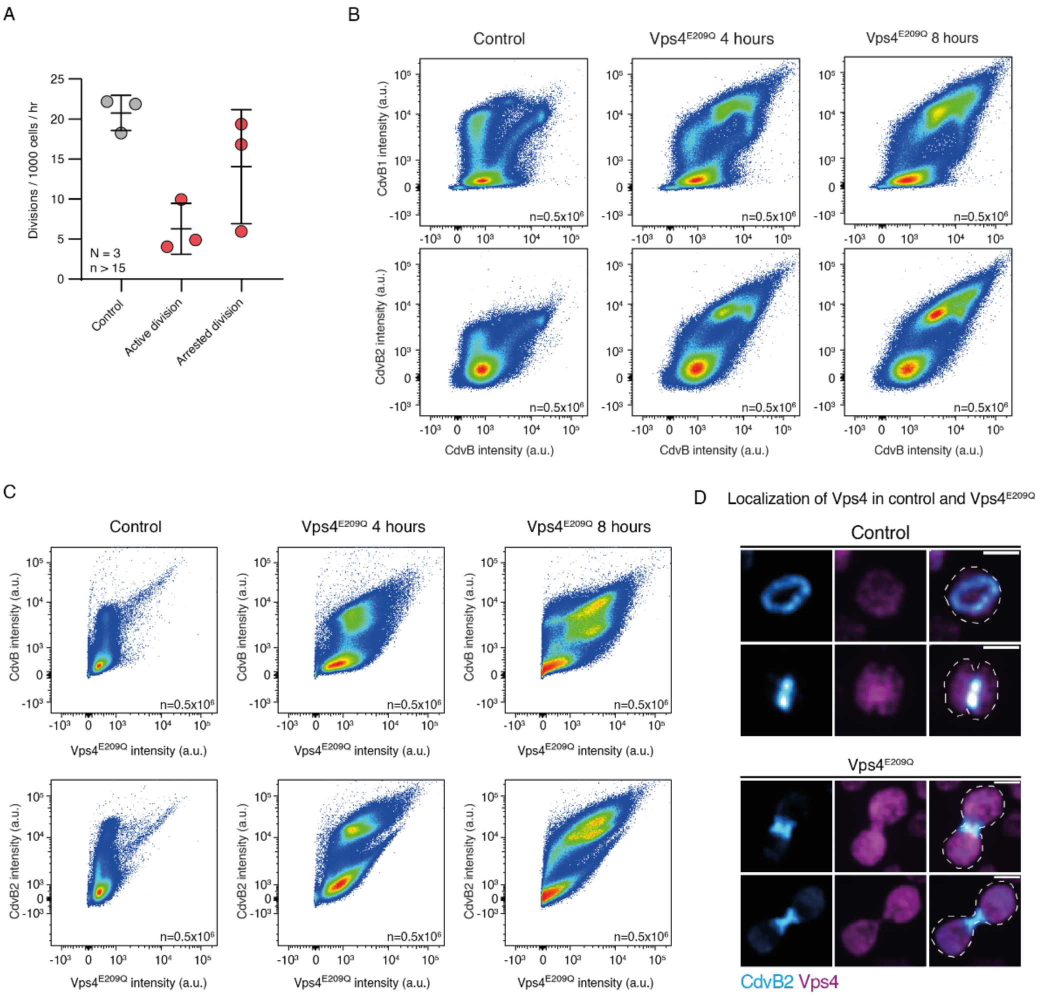

Figure S2. Vps4 is required for cell division. **(A)** Quantification of cell divisions from live imaging of MW001 control and Vps4^{E209Q} (N = 3). **(B)** Representative cytograms of CdvB protein levels vs CdvB1 and CdvB2 in MW001 control and Vps4^{E209Q} (N = 3). **(C)** Representative cytograms of Vps4^{E209Q} intensity versus CdvB and CdvB2 in MW001 control and Vps4^{E209Q} (N = 3). **(D)** Fluorescence imaging of Vps4 localization in control and Vps4^{E209Q} cells. Scale bar = 1 µm.

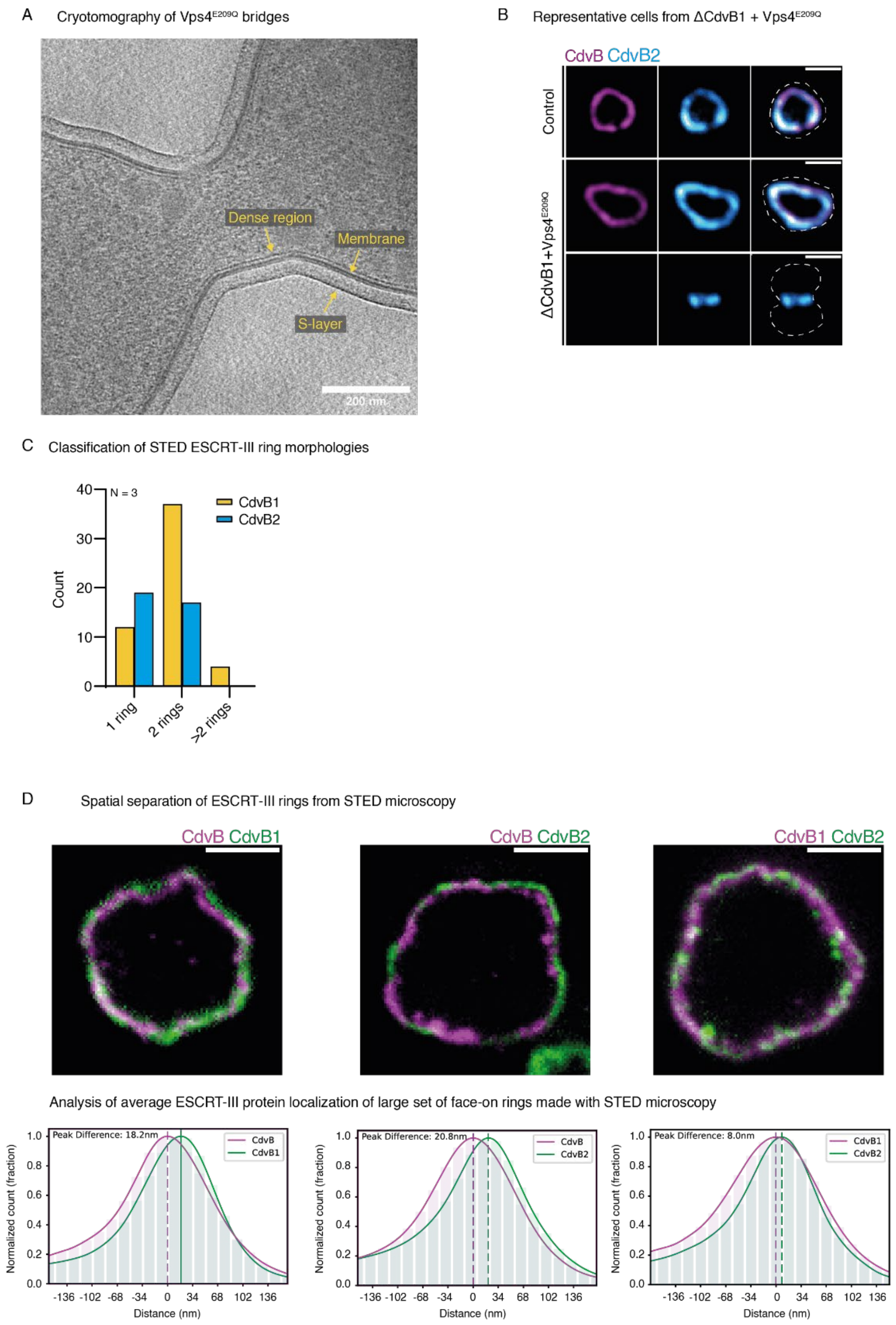

Figure S3. CdvB, CdvB1 and CdvB2 have different relative localizations and roles. **(A)** Cryotomography of Vps4^{E209Q} arrested dumbbell-shaped cells. **(B)** Representative spinning disc microscopy images of MW001 control and ΔCdvB1+Vps4^{E209Q} cells (N = 3). **(C)** Classification of ring morphologies from STED microscopy from a large set of images of non-

constricted cells retaining a CdvB ring. **(D)** Representative rings when viewed face on by two-colour STED microscopy. Scale bar = 0.5 µm (N = 3). Graph shows quantification of averaged ring radii (two per diameter) taken from these data (N = 3, n = 115). Figure presented in two-colour for clarity of visualization.

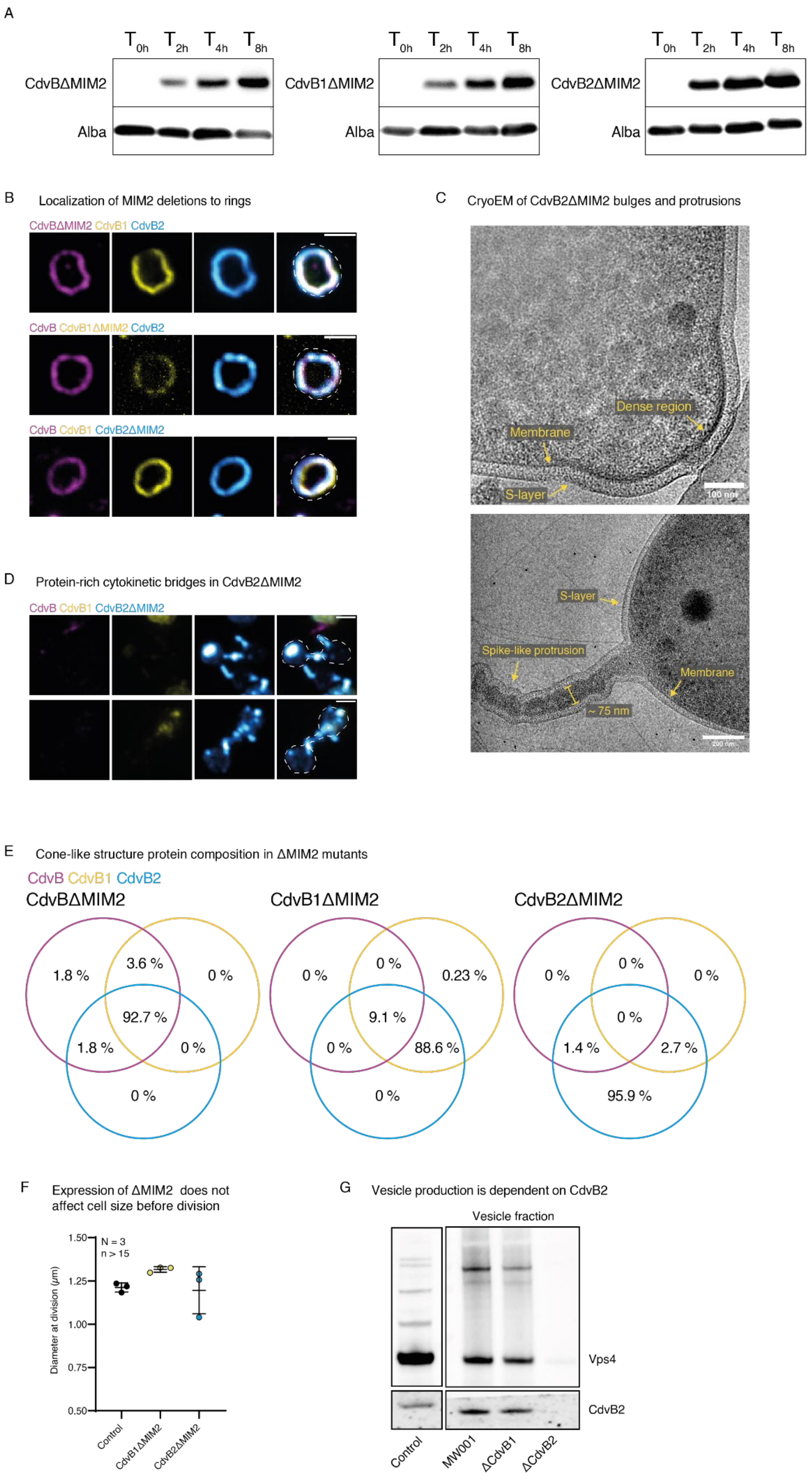
A
T0h T2h T4h T8h
CdvBΔMIM2
Alba
CdvB1ΔMIM2
Alba
CdvB2ΔMIM2
Alba
B
Localization of MIM2 deletions to rings
CdvBΔMIM2 CdvB1 CdvB2
CdvB CdvB1ΔMIM2 CdvB2
CdvB CdvB1 CdvB2ΔMIM2
C
CryoEM of CdvB2ΔMIM2 bulges and protrusions
Dense region
Membrane
S-layer
100 nm
S-layer
Spike-like protrusion
Membrane
~ 75 nm
200 nm
D
Protein-rich cytokinetic bridges in CdvB2ΔMIM2
CdvB CdvB1 CdvB2ΔMIM2
E
Cone-like structure protein composition in ΔMIM2 mutants
CdvB CdvB1 CdvB2
CdvBΔMIM2
1.8 %
3.6 %
0 %
92.7 %
1.8 %
0 %
0 %
CdvB1ΔMIM2
0 %
0 %
0.23 %
9.1 %
0 %
88.6 %
0 %
CdvB2ΔMIM2
0 %
0 %
0 %
0 %
1.4 %
2.7 %
95.9 %
F
Expression of ΔMIM2 does not affect cell size before division
N = 3
n > 15
Diameter at division (μm)
1.50
1.25
1.00
0.75
0.50
Control
CdvB1ΔMIM2
CdvB2ΔMIM2
G
Vesicle production is dependent on CdvB2
Vesicle fraction
Vps4
CdvB2
Control
MW001
ΔCdvB1
ΔCdvB2

Figure S4. Sequential remodelling of CdvB1 and CdvB2 is required for proper constriction and cell division. **(A)** Western blot of ΔMIM2 mutant induction over time, with Alba protein serving as a loading control (N = 3). **(B)** Representative spinning disc microscopy images of CdvBΔMIM2, CdvB1ΔMIM2 and CdvB2ΔMIM2 rings. Scale bar = 1 µm. **(C)** CryoEM of conical CdvB2ΔMIM2 localized bulges and spike-like protrusion. **(D)** Representative examples of daughter cells connected by narrow bridges with high levels of CdvB2ΔMIM2 (N = 3). **(E)** Quantification of CdvB protein presence in localized accumulation present in ΔMIM2 strains (N = 3). **(F)** Quantification of cell size of MW001 control and ΔMIM2 strains from live imaging (N = 3). **(G)** Western blot of MW001 control and vesicles fractions of MW001, ΔCdvB1 and ΔCdvB2, with staining against Vps4 and CdvB2.

Heatmaps exploring the impact of the filament energy on the constriction mechanism

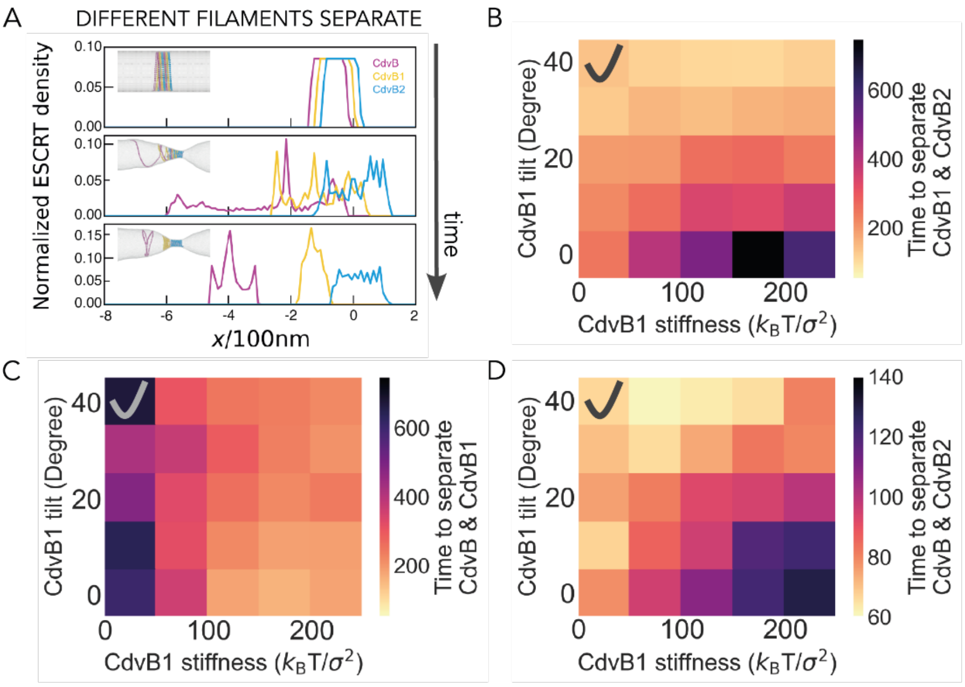

Figure S5. Typical snapshots along the spontaneous filament separation trajectory of CdvB, CdvB1, CdvB2 in the absence of Vps4 **(A)** and the time to separate CdvB1 and CdvB2 **(B)**, CdvB and CdvB1 **(C)**, CdvB and CdvB2 **(D)** (in the unit of t_0) as a function of CdvB1 stiffness and tilt. Data is averaged from 5 independent simulations. $k_{CdvB} = k_{CdvB2} = 250\ k_B T/\sigma^2$. In (A), $k_{CdvB1} = 200\ k_B T/\sigma^2$, $\theta_{CdvB1} = 40°$. The biologically relevant parameter regime (CdvB1 soft and tilted) is indicated with a tick label in panels B-D.

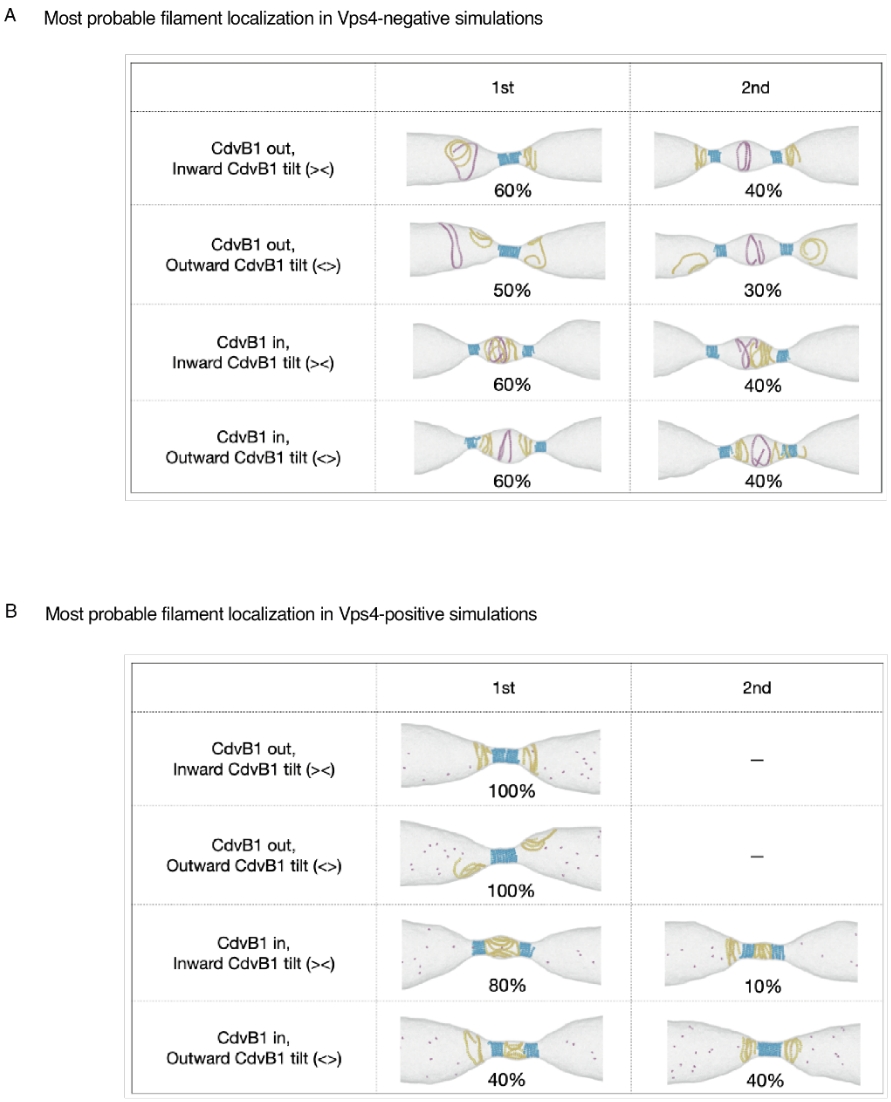

Figure S6. Representative snapshots of the first and second probable filament localization in the absence of Vps4 **(A)** and in the presence of Vps4 **(B)**. The most probable configurations are displayed in Fig. 5A.

A Snapshots of a simulation with CdvB1 - CdvB2 - CdvB1 patterning inside a tube

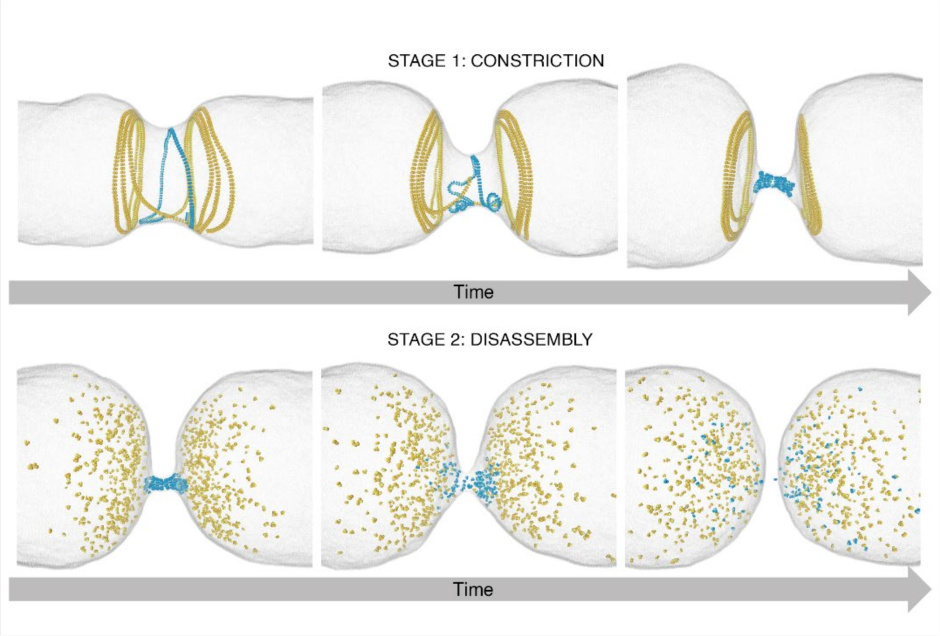

Figure S7. Snapshots of a simulation with CdvB1 - CdvB2 - CdvB1 patterning inside a tube with R_{tube} = 27σ, where tension is kept low by imposing zero pressure along the tube axis. The filaments were not supplied with all the energy instantaneously, but instead slowly transformed via transitioning one random subunit within the filament at a time. The initial filament state for both CdvB1 and CdvB2 was $R_{CdvB1} = R_{CdvB2} = 27\sigma$, $k_{CdvB1} = k_{CdvB2} = 400\, k_BT/\sigma^2$, and $\tau_{CdvB1} = \tau_{CdvB2}$= 0°. CdvB1 then transformed to a new state in which $R_{CdvB1} = 0.6R_{tube}$, $k_{CdvB1} = 400\, k_BT/\sigma^2$, and τ_{CdvB1}= 90°, and CdvB2 to $R_{CdvB2} = 1.75\ \sigma$, $k_{CdvB2} = 400\, k_BT/\sigma^2$, and τ_{CdvB2}= 0°. This led to CdvB1 forming funnels on each side of CdvB2 and CdvB2 constricting the membrane neck to a tube. Upon disassembly of CdvB1 and then CdvB2, the cell divided.

Table S1. Overview of the STED DyMIN settings for the ESCRT-III proteins CdvB, CdvB1 and CdvB2 in either the 640nm channel (protein labelled with Abberior STAR 635) or the 561nm channel (protein labelled with Abberior STAR 580). Laser intensities are indicated in percentages of the laser heads, where the 640 nm laser power is 1mW and the 561nm laser has 200μW power. The dwell time of a single line was consistently 13.5 μs.

640nm channel	561nm channel	Line steps			Intensity Confocal laser (%)			Intensity STED doughnut (%)	
		Confocal	Low int. STED	High int. STED	Confocal	Low int. STED	High int. STED	Low int. STED	High int. STED
CdvB		5	6	19	0.4	0.5	1.1	4.6	45
	CdvB	2	8	8	7.8	13	20	15	90
CdvB1		3	5	18	0.2	0.45	1.1	3.6	45
	CdvB1	1	4	5	3	4	15	12	90
CdvB2		3	5	18	0.2	0.45	1.1	3.6	45
	CdvB2	2	10	8	17	20	25	15	90

Table S2. List of secondary antibodies used for STED experiments with wavelengths and Abberior catalogue numbers.

Abberior Star 580 goat anti-rabbit IgG	ST580-1002-500UG
Abberior Star 635 goat anti-rabbit IgG	ST635-1002-500UG
Abberior Star 580 goat anti-chicken IgY	ST580-1005-500UG
Abberior Star 635P goat anti-chicken IgY	ST635-1005-500UG
Abberior Star 580 goat anti-guinea pig IgG	ST580-1006-500UG
Abberior Star 635 goat anti-guinea pig IgG	ST635-1006-500UG

Table S3. List of primary antibodies, host animal and supplier.

Antibody (animal)	Supplier
Anti-CdvB (rabbit)	Lab generated
Anti-CdvB1 (chicken)	Lab generated
Anti-CdvB2 (guinea pig)	Lab generated
Anti-Vps4 (rabbit)	Lab generated
Anti-HA (mouse)	ThermoFisher, 26183
Anti-His (mouse)	Abcam, ab18184

Table S4. List of secondary antibodies, host animal, ThermoFisher catalogue numbers and wavelengths.

ThermoFisher	Anti-Rabbit	Anti-Chicken	Anti-Guinea pig	Anti-Mouse
Alexa Fluor 405	A48254	A48260	- - -	A31553
Alexa Fluor 488	A11034	A11039	A11073	A10631
Alexa Fluor 546	A11035	A11040	A11074	A11030
Alexa Fluor 647	A21245	A21449	A21450	A21235

Movie S1. Cryotomogram of a division bridge in Vps4^{E209Q} arrested dumbbell-shaped cells. A slice is shown in **Fig. S3A**. An electron-dense region lines the membrane, correlating well with observed ESCRT-III and Vps4 localizations.

Movie S2. Live cell imaging of *S. acidocaldarius* expressing CdvB2ΔMIM2, displaying rigid membrane protrusions.

Movie S3. Coarse-grained molecular dynamics simulations of Vps4+ conditions described in **Fig. 5** and **Fig. 6**. **(A)** CdvB1 polymer outside of CdvB2 polymer, inward CdvB1 tilt. **(B)** CdvB1 polymer inside of CdvB2 polymer, inward CdvB1 tilt.

REFERENCES

1. B. Junglas, S. T. Huber, T. Heidler, L. Schlösser, D. Mann, R. Hennig, M. Clarke, N. Hellmann, D. Schneider, C. Sachse, PspA adopts an ESCRT-III-like fold and remodels bacterial membranes. *Cell* **184**, 3674–3688.e18 (2021).

2. J. Liu, M. Tassinari, D. P. Souza, S. Naskar, J. K. Noel, O. Bohuszewicz, M. Buck, T. A. Williams, B. Baum, H. H. Low, Bacterial Vipp1 and PspA are members of the ancient ESCRT-III membrane-remodeling superfamily. *Cell* **184**, 3660–3673.e18 (2021).

3. M. Vietri, M. Radulovic, H. Stenmark, The many functions of ESCRTs. *Nat. Rev. Mol. Cell Biol.* **21**, 25–42 (2020).

4. J. H. Hurley, P. I. Hanson, Membrane budding and scission by the ESCRT machinery: It's all in the neck. *Nat. Rev. Mol. Cell Biol.* **11**, 556–566 (2010).

5. Y. Caspi, C. Dekker, Dividing the archaeal way: The ancient Cdv cell-division machinery. *Front Microbiol.* **9**, 174 (2018).

6. J. McCullough, A. Frost, W. I. Sundquist, Structures, functions, and dynamics of ESCRT-III/Vps4 membrane remodeling and fission complexes. *Annu. Rev. Cell Dev. Biol.* **34**, 85–109 (2018).

7. J. McCullough, A. K. Clippinger, N. Talledge, M. L. Skowyra, M. G. Saunders, T. V. Naismith, L. A. Colf, P. Afonine, C. Arthur, W. I. Sundquist, P. I. Hanson, A. Frost, Structure and membrane remodeling activity of ESCRT-III helical polymers. *Science* **350**, 1548–1551 (2015).

8. Q.-T. Shen, A. L. Schuh, Y. Zheng, K. Quinney, L. Wang, M. Hanna, J. C. Mitchell, M. S. Otegui, P. Ahlquist, Q. Cui, A. Audhya, Structural analysis and modeling reveals new mechanisms governing ESCRT-III spiral filament assembly. *J. Cell Biol.* **206**, 763–777 (2014).

9. R. Pires, B. Hartlieb, L. Signor, G. Schoehn, S. Lata, M. Roessle, C. Moriscot, S. Popov, A. Hinz, M. Jamin, V. Boyer, R. Sadoul, E. Forest, D. I. Svergun, H. G. Göttlinger, W. Weissenhorn, A crescent-shaped ALIX dimer targets ESCRT-III CHMP4 filaments. *Structure* **17**, 843–856 (2009).

10. N. Chiaruttini, A. Roux, Dynamic and elastic shape transitions in curved ESCRT-III filaments. *Curr. Opin. Cell Biol.* **47**, 126–135 (2017).

11. P. I. Hanson, R. Roth, Y. Lin, J. E. Heuser, Plasma membrane deformation by circular arrays of ESCRT-III protein filaments. *J. Cell Biol.* **180**, 389–402 (2008).

12. W. M. Henne, N. J. Buchkovich, Y. Zhao, S. D. Emr, The endosomal sorting complex ESCRT-II mediates the assembly and architecture of ESCRT-III helices. *Cell* **151**, 356–371 (2012).

13. A.-K. Pfitzner, V. Mercier, X. Jiang, J. Moser von Filseck, B. Baum, A. Šarić, A. Roux, An ESCRT-III polymerization sequence drives membrane deformation and fission. *Cell* **182**, 1140–1155.e18 (2020).

14. B. E. Mierzwa, N. Chiaruttini, L. Redondo-Morata, J. Moser von Filseck, J. König, J. Larios, I. Poser, T. Müller-Reichert, S. Scheuring, A. Roux, D. W. Gerlich, Dynamic subunit turnover in ESCRT-III assemblies is regulated by Vps4 to mediate membrane remodelling during cytokinesis. *Nat. Cell Biol.* **19**, 787–798 (2017).

15. M. A. Y. Adell, S. M. Migliano, S. Upadhyayula, Y. S. Bykov, S. Sprenger, M. Pakdel, G. F. Vogel, G. Jih, W. Skillern, R. Behrouzi, M. Babst, O. Schmidt, M. W. Hess, J. A. Briggs, T. Kirchhausen, D. Teis, Recruitment dynamics of ESCRT-III and Vps4 to endosomes and implications for reverse membrane budding. *eLife* **6**, e31652 (2017).

16. A. K. Cada, M. R. Pavlin, J. P. Castillo, A. B. Tong, K. P. Larsen, X. Ren, A. L. Yokom, F.-C. Tsai, J. V. Shiah, P. M. Bassereau, C. J. Bustamante, J. H. Hurley, Friction-driven membrane scission by the human ESCRT-III proteins CHMP1B and IST1. *Proc. Natl. Acad. Sci.* **119**, e2204536119 (2022).

17. J. McCullough, W. I. Sundquist, Membrane remodeling: ESCRT-III filaments as molecular garrotes. *Curr. Biol.* **30**, R1425–R1428 (2020).

18. B. Meadowcroft, I. Palaia, A.-K. Pfitzner, A. Roux, B. Baum, A. Šarić, Mechanochemical rules for membrane-reshaping composite filaments. *bioRxiv*, 2022.05.10.490642 (2022).

19. L. Harker-Kirschneck, B. Baum, A. Šarić, Changes in ESCRT-III filament geometry drive membrane remodelling and fission in silico. *BMC Biol.* **17**, 82 (2019).

20. K. Zaremba-Niedzwiedzka, E. F. Caceres, J. H. Saw, D. Bäckström, L. Juzokaite, E. Vancaester, K. W. Seitz, K. Anantharaman, P. Starnawski, K. U. Kjeldsen, M. B. Stott, T. Nunoura, J. F. Banfield, A. Schramm, B. J. Baker, A. Spang, T. J. G. Ettema, Asgard archaea illuminate the origin of eukaryotic cellular complexity. *Nature* **541**, 353–358 (2017).

21. C. J. Castelle, J. F. Banfield, Major new microbial groups expand diversity and alter our understanding of the tree of life. *Cell* **172**, 1181–1197 (2018).

22. T. Hatano, S. Palani, D. Papatziamou, R. Salzer, D. P. Souza, D. Tamarit, M. Makwana, A. Potter, A. Haig, W. Xu, D. Townsend, D. Rochester, D. Bellini, H. M. A. Hussain, T. J. G. Ettema, J. Löwe, B. Baum, N. P. Robinson, M. Balasubramanian, Asgard archaea shed light on the evolutionary origins of the eukaryotic ubiquitin-ESCRT machinery. *Nat. Commun.* **13**, 3398 (2022).

23. E. A. Pelve, A.-C. Lindås, W. Martens-Habbena, J. R. de la Torre, D. A. Stahl, R. Bernander, Cdv-based cell division and cell cycle organization in the thaumarchaeon *Nitrosopumilus maritimus*. *Mol. Microbiol.* **82**, 555–566 (2011).

24. R. Y. Samson, T. Obita, S. M. Freund, R. L. Williams, S. D. Bell, A role for the ESCRT system in cell division in archaea. *Science* **322**, 1710–1713 (2008).

25. A.-C. Lindås, E. A. Karlsson, M. T. Lindgren, T. J. G. Ettema, R. Bernander, A unique cell division machinery in the archaea. *Proc. Natl. Acad. Sci. U.S.A.* **105**, 18942–18946 (2008).

26. A. Blanch Jover, N. De Franceschi, D. Fenel, W. Weissenhorn, C. Dekker, The archaeal division protein CdvB1 assembles into polymers that are depolymerized by CdvC. *FEBS Lett.* **596**, 958–969 (2022).

27. G. T. Risa, F. Hurtig, S. Bray, A. E. Hafner, L. Harker-Kirschneck, P. Faull, C. Davis, D. Papatziamou, D. R. Mutavchiev, C. Fan, L. Meneguello, A. A. Pulschen, G. Dey, S. Culley, M. Kilkenny, D. P. Souza, L. Pellegrini, R. A. M. de Bruin, R. Henriques, A. P. Snijders, A. Šarić, A.-C. Lindås, N. P. Robinson, B. Baum, The proteasome controls ESCRT-III–mediated cell division in an archaeon. *Science* **369**, eaaz2532 (2020).

28. M. Babst, B. Wendland, E. J. Estepa, S. D. Emr, The Vps4p AAA ATPase regulates membrane association of a Vps protein complex required for normal endosome function. *EMBO J.* **17**, 2982–2993 (1998).

29. T. Gristwood, I. G. Duggin, M. Wagner, S. V. Albers, S. D. Bell, The sub-cellular localization of *Sulfolobus* DNA replication. *Nucleic Acids Res.* **40**, 5487–5496 (2012).

30. R. Bernander, The cell cycle of *Sulfolobus*. *Mol. Microbiol.* **66**, 557–562 (2007).

31. J. Wuarin, P. Nurse, Regulating S phase: CDKs, licensing and proteolysis. *Cell* **85**, 785–787 (1996).

32. A. C. Porter, Preventing DNA over-replication: A Cdk perspective. *Cell Div.* **3**, 3 (2008).

33. J. F. X. Diffley, Regulation of early events in chromosome replication. *Curr. Biol.* **14**, R778–R786 (2004).

34. A. A. Pulschen, D. R. Mutavchiev, S. Culley, K. N. Sebastian, J. Roubinet, M. Roubinet, G. T. Risa, M. van Wolferen, C. Roubinet, U. Schmidt, G. Dey, S.-V. Albers, R. Henriques, B. Baum, Live imaging of a hyperthermophilic archaeon reveals distinct roles for two ESCRT-III homologs in ensuring a robust and symmetric division. *Curr. Biol.* **30**, 2852–2859.e4 (2020).

35. L. Harker-Kirschneck, A. E. Hafner, T. Yao, C. Vanhille-Campos, X. Jiang, A. Pulschen, F. Hurtig, D. Hryniuk, S. Culley, R. Henriques, B. Baum, A. Šarić, Physical mechanisms of ESCRT-III-driven cell division. *Proc. Natl. Acad. Sci. U.S.A.* **119**, e2107763119 (2022).

36. S. Lata, G. Schoehn, A. Jain, R. Pires, J. Piehler, H. G. Gőttlinger, W. Weissenhorn, Helical structures of ESCRT-III are disassembled by VPS4. *Science* **321**, 1354–1357 (2008).

37. S. Saksena, J. Wahlman, D. Teis, A. E. Johnson, S. D. Emr, Functional reconstitution of ESCRT-III assembly and disassembly. *Cell* **136**, 97–109 (2009).

38. B. Yang, G. Stjepanovic, Q. Shen, A. Martin, J. H. Hurley, Vps4 disassembles an ESCRT-III filament by global unfolding and processive translocation. *Nat. Struct. Mol. Biol.* **22**, 492–498 (2015).

39. M. J. Dobro, R. Y. Samson, Z. Yu, J. McCullough, H. J. Ding, P. L.-G. Chong, S. D. Bell, G. J. Jensen, Electron cryotomography of ESCRT assemblies and dividing *Sulfolobus* cells suggests that spiraling filaments are involved in membrane scission. *Mol. Biol. Cell* **24**, 2319–2327 (2013).

40. R. Kojima, T. Obita, K. Onoue, M. Mizuguchi, Structural fine-tuning of MIT-interacting motif 2 (MIM2) and allosteric regulation of ESCRT-III by Vps4 in yeast. *J. Mol. Biol.* **428**, 2392–2404 (2016).

41. D. M. Wenzel, D. R. Mackay, J. J. Skalicky, E. L. Paine, M. S. Miller, K. S. Ullman, W. I. Sundquist, Comprehensive analysis of the human ESCRT-III-MIT domain interactome reveals new cofactors for cytokinetic abscission. *bioRxiv*, 2022.02.09.477148 (2022).

42. C. Caillat, S. Maity, N. Miguet, W. H. Roos, W. Weissenhorn, The role of VPS4 in ESCRT-III polymer remodeling. *Biochem. Soc. Trans.* **47**, 441–448 (2019).

43. K. Azad, D. Guilligay, C. Boscheron, S. Maity, N. D. Franceschi, G. Sulbaran, G. Effantin, H. Wang, J.-P. Kleman, P. Bassereau, G. Schoehn, W. H. Roos, A. Desfosses, W. Weissenhorn, Structural basis of CHMP2A-CHMP3 ESCRT-III polymer assembly and membrane cleavage. *bioRxiv*, 2022.04.12.487901 (2022).

44. J. Liu, R. Gao, C. Li, J. Ni, Z. Yang, Q. Zhang, H. Chen, Y. Shen, Functional assignment of multiple ESCRT-III homologs in cell division and budding in *Sulfolobus islandicus*. *Mol. Microbiol.* **105**, 540–553 (2017).

45. I. Goliand, S. Adar-Levor, I. Segal, D. Nachmias, T. Dadosh, M. M. Kozlov, N. Elia, Resolving ESCRT-III spirals at the intercellular bridge of dividing cells using 3D STORM. *Cell Rep.* **24**, 1756–1764 (2018).

46. J. Guizetti, L. Schermelleh, J. Mäntler, S. Maar, I. Poser, H. Leonhardt, T. Müller-Reichert, D. W. Gerlich, Cortical constriction during abscission involves helices of ESCRT-III–dependent filaments. *Science* **331**, 1616–1620 (2011).

47. J. G. Carlton, J. Martin-Serrano, Parallels between cytokinesis and retroviral budding: A role for the ESCRT machinery. *Science* **316**, 1908–1912 (2007).

48. M. Wagner, S. Berkner, M. Ajon, A. J. M. Driessen, G. Lipps, S.-V. Albers, Expanding and understanding the genetic toolbox of the hyperthermophilic genus *Sulfolobus*. *Biochem. Soc. Trans.* **37**, 97–101 (2009).

49. F. N. Mol, R. Vlijm, Automated STED nanoscopy for high-throughput imaging of cellular structures. *bioRxiv*, 2022.09.29.510126 (2022).

50. F. N. Mol, R. Vlijm, Ring-colocalization: Analysis of dual-color images of ring-like structures (2022); https://doi.org/10.5281/zenodo.7307223.

51. P. Thevenaz, U. E. Ruttimann, M. Unser, A pyramid approach to subpixel registration based on intensity. *IEEE Trans. Image Process.* **7**, 27–41 (1998).

52. C. J. Russo, S. Scotcher, M. Kyte, A precision cryostat design for manual and semi-automated cryo-plunge instruments. *Rev. Sci. Instrum.* **87**, 114302 (2016).

53. M. D. Abràmoff, P. J. Magalhães, S. J. Ram, Image processing with ImageJ. *Biophotonics Int.* **11**, 36–42 (2004).

54. W. J. H. Hagen, W. Wan, J. A. G. Briggs, Implementation of a cryo-electron tomography tilt-scheme optimized for high resolution subtomogram averaging. *J. Struct. Biol.* **197**, 191–198 (2017).

55. D. N. Mastronarde, Automated electron microscope tomography using robust prediction of specimen movements. *J. Struct. Biol.* **152**, 36–51 (2005).

56. J. R. Kremer, D. N. Mastronarde, J. R. McIntosh, Computer visualization of three-dimensional image data using IMOD. *J. Struct. Biol.* **116**, 71–76 (1996).

57. A. Ji, F. Jj, Tomo3D 2.0–Exploitation of advanced vector extensions (AVX) for 3D reconstruction. *J. Struct. Biol.* **189**, 147–152 (2015).

58. G. Tang, L. Peng, P. R. Baldwin, D. S. Mann, W. Jiang, I. Rees, S. J. Ludtke, EMAN2: An extensible image processing suite for electron microscopy. *J. Struct. Biol.* **157**, 38–46 (2007).

59. H. Yuan, C. Huang, J. Li, G. Lykotrafitis, S. Zhang, One-particle-thick, solvent-free, coarse-grained model for biological and biomimetic fluid membranes. *Phys. Rev. E Stat. Nonlin. Soft Matter Phys.* **82**, 011905 (2010).

60. A. P. Thompson, H. M. Aktulga, R. Berger, D. S. Bolintineanu, W. M. Brown, P. S. Crozier, P. J. in’t Veld, A. Kohlmeyer, S. G. Moore, T. D. Nguyen, R. Shan, M. J. Stevens, J. Tranchida, C. Trott, S. J. Plimpton, LAMMPS–A flexible simulation tool for particle-based materials modeling at the atomic, meso, and continuum scales. *Comput. Phys. Commun.* **271**, 108171 (2022).

61. J. Lafaurie-Janvore, P. Maiuri, I. Wang, M. Pinot, J.-B. Manneville, T. Betz, M. Balland, M. Piel, ESCRT-III assembly and cytokinetic abscission are induced by tension release in the intercellular bridge. *Science* **339**, 1625–1629 (2013).
